# Regulatory B cell-related gene signature predicts prognosis and immune landscape in head and neck squamous cell carcinoma

**DOI:** 10.3389/fimmu.2026.1739076

**Published:** 2026-04-10

**Authors:** Junyan He, Keling Pang, Zhigang Zhou, Chaolin Yang, Yi Chen, Sha He, Fujue Wang, Pian Li

**Affiliations:** 1 Department of Oncology, The First Affiliated Hospital, Hengyang Medical School, University of South China, Hengyang, China; 2 Department of Radiation Oncology, The First Affiliated Hospital of Guangxi Medical University, Nanning, China; 3 Changde Hospital, Xiangya School of Medicine, Central South University, The First People’s Hospital of Changde City, Changde, China; 4 Department of Hematology, The First Affiliated Hospital, Hengyang Medical School, University of South China, Hengyang, Hunan, China; 5 State Key Laboratory of Biotherapy, West China Hospital, Sichuan University, Chengdu, Sichuan, China; 6 Collaborative Innovation Center for Biotherapy, West China Hospital, Sichuan University, Chengdu, Sichuan, China

**Keywords:** head and neck squamous cell carcinoma (HNSCC), immune infiltration, prognostic model, regulatory B cells (Bregs), therapeutic strategies

## Abstract

**Background:**

Regulatory B cells (Bregs) are critical in regulating immune responses and fostering immune tolerance in various cancers; however, their role in head and neck squamous cell carcinoma (HNSCC) is unclear. This study examined the function of Breg-related genes in HNSCC and their possible prognostic and therapeutic implications.

**Methods:**

The Cancer Genome Atlas (TCGA)-HNSCC training cohort was used to establish a prognostic signature for Breg-related genes by applying consensus clustering, univariate Cox regression, least absolute shrinkage and selection operator (LASSO) Cox regression, and multivariate Cox regression analyses. Validation cohorts from the TCGA and Gene Expression Omnibus (GEO) databases were used to assess the robustness of the model. This study investigated the associations among the signature and several clinicopathological features, expression of immune checkpoints, tumor mutation burden (TMB), and sensitivity to pharmacological agents. The underlying mechanisms were examined using weighted gene co-expression network analysis (WGCNA) and gene set enrichment analysis (GSEA). Additionally, various techniques, including ESTIMATE, were used to assess immune infiltration. Functional experiments and transcriptome sequencing were conducted to investigate the role of oxidized low-density lipoprotein receptor 1 (OLR1) gene.

**Results:**

The analysis identified an eight-gene Breg-related prognostic signature that demonstrated robust predictive power across cohorts. High-risk patients exhibited significantly poorer survival, reduced immune cell infiltration, and lower immune molecule expression. The prognostic accuracy was further improved by integrating the risk score with TMB or clinicopathological features. Functional analyses revealed strong associations with immune-related pathways. Moreover, the signature was reported as a potential biomarker for predicting immunotherapy response and drug sensitivity. Furthermore, OLR1, the most essential gene of the signature, was found to be oncogenic and linked to immune evasion in HNSCC.

**Conclusions:**

The Breg-related gene signature provides an effective prognostic tool for patients with HNSCC, reflects the immune landscape and TMB, and may direct personalized therapeutic approaches, such as immunotherapy.

## 1 Introduction

Head and neck squamous cell carcinoma (HNSCC) is a prevalent global health concern. It is one of the most common malignant tumors originating from the mucosal epithelium of the oral cavity, pharynx, and larynx. According to epidemiological studies, HNSCC results in more than 600,000 new cases annually worldwide, disproportionately affecting low- and middle-income countries due to limited access to early screening and comprehensive care (1, 2). Despite advancements in multimodal treatments such as surgery, radiotherapy, and chemotherapy, the overall survival (OS) rates for patients with advanced or recurrent HNSCC remain dismal, with five-year survival rates displaying minimal improvement over the previous decades (3, 4). These trends highlight the urgent need for novel diagnostic, prognostic, and therapeutic techniques to improve patient outcomes.

The tumor microenvironment (TME) and the complex interactions between cancer cells and the host immune system have recently become the focus of oncological research (4, 5). Some patients with recurrent or metastatic disease have experienced considerable clinical advantage from immunotherapy, particularly immune checkpoint inhibition, which has demonstrated promise as a therapeutic option for HNSCC. However, the overall response rate is still modest, and predictive biomarkers for immunotherapeutic efficacy are incompletely characterized (3, 4, 6). The molecular and immunological heterogeneity of HNSCC complicates treatment selection and underscores the limitations of the current one-size-fits-all approaches. Immune evasion mechanisms, such as alterations in immune cell infiltration and immune checkpoint expression, play a role in therapeutic resistance and disease progression (5, 7).

Regulatory B cells (Bregs) have drawn more attention than other immune cell subsets implicated in cancer biology, due to their strong immunosuppressive properties. Bregs primarily function by secreting anti-inflammatory cytokines, such as interleukin (IL)-10, transforming growth factor-beta (TGF-β) and IL-35, which regulate both innate and adaptive immune responses (8, 9). Bregs were first identified in autoimmunity and transplantation tolerance, but they are now recognized as critical in tumor immune evasion, which promotes tumor progression by suppressing effector T cells and enhancing the proliferation of regulatory T cells (10). Although the importance of Bregs has been established in a number of cancers (11–14), their precise functions, phenotypic markers, and transcriptomic profiles in HNSCC remain mostly unexplored, which represents a significant gap in the understanding of the immunobiology of HNSCC.

This study systematically investigated Breg-associated gene expression in HNSCC using integrative bioinformatics and comprehensive transcriptomic data from The Cancer Genome Atlas (TCGA) and Gene Expression Omnibus (GEO) databases. This research aimed to develop and validate a clinically valuable Breg-related gene signature for prognostic assessment and treatment guidance by using unsupervised clustering, prognostic modeling, and immune infiltration analyses. It also investigated immunotherapy and drug sensitivity prediction, aiming to use Breg-associated indicators for personalized oncology treatment to support the move towards individualized medicine.

## 2 Materials and methods

### 2.1 Data sources and processing

A set of 27 Breg-related genes was obtained from a previously published study (13), which systematically identified regulatory B cell-associated genes based on their well-characterized functional involvement in immunosuppression, including IL−10–mediated signaling, immune tolerance, and the regulation of adaptive immune responses, rather than relying solely on phenotypic surface markers. Gene expression profiles and clinical data for patients with HNSCC (n = 493) were obtained from the TCGA database (https://portal.gdc.cancer.gov/). The baseline clinicopathological characteristics of patients in the TCGA-HNSCC cohort are summarized in **Supplementary Table 1**. The patients were randomly assigned to the training and test cohorts in a 1:1 ratio. Meanwhile, GSE41613 (n = 96) and GSE65858 (n = 267) datasets were identified as the external validation cohorts. Patients having an OS longer than 30 days and complete gene expression data were selected for analysis. Immunotherapy response was examined using the IMvigor210 (http://research-pub.gene.com/IMvigor210CoreBiologies/), GSE78220, and GSE91061 datasets (15). The differential expression of the oxidized low-density lipoprotein receptor 1 (OLR1) gene was analyzed using the TCGA pan-cancer cohort and numerous GEO datasets, including GSE23558, GSE30784, GSE184616, and GSE34106. All GSE datasets were sourced from the GEO database (https://www.ncbi.nlm.nih.gov/geo/).

### 2.2 Consensus clustering identified Breg-related clusters

Breg-related genes in patients with HNSCC were analyzed using the ‘ConsensusClusterPlus’ package (16) to identify distinct Breg-related patterns and categorize patients into subtypes according to gene expression levels. The optimal count of clusters (k) was determined by evaluating the cumulative distribution function (CDF) curve and analyzing the relative variation in its area, represented by the Δ area plot.

### 2.3 Immune infiltration and immune checkpoint expression analyses

The ‘ESTIMATE’ R package (17) was used to evaluate immune cell infiltration and characterize the TME by computing immunological, stromal, and ESTIMATE scores. Single-sample gene set enrichment analysis (GSEA) was used to quantify the infiltration of 28 immune cell subsets across different clusters or risk groups using the ‘GSVA’ R package (18). Several immune cell deconvolution algorithms were used, including XCell, TIMER, QUANTISEQ, MCPCOUNTER, CIBERSORT-ABS, CIBERSORT, to validate alterations in the immune landscape (19). Moreover, immune checkpoint expression was assessed and compared between the two risk groups.

### 2.4 Differential gene expression and functional enrichment analyses

GSEA was used to compare gene ontology (GO) enrichment between groups C1 and C2. Differentially expressed genes (DEGs) between Cluster C1 and Cluster C2 were identified using the Wilcoxon rank-sum test (20), with the criteria of |Fold Change (FC)| > 1.5 and *p*-value < 0.05. The volcano plot of these genes was generated using the ‘ggplot’ package in R (https://CRAN.R-project.org/package=ggplot2). Functional enrichment analyses, encompassing GO and Kyoto encyclopedia of genes and genomes (KEGG), were conducted using the ‘clusterProfiler’ R package (21).

### 2.5 Development and validation of the Breg-related gene prognostic signature

A univariate Cox regression analysis was first performed on the TCGA training cohort to assess the prognostic importance of Breg-related genes in HNSCC. Subsequently, least absolute shrinkage and selection operator (LASSO) regression through the ‘glmnet’ R package (22) and stepwise multivariate Cox regression analysis were used to identify prognostically important genes. The regression coefficients (coef) and hazard ratios for each gene were derived from the multivariate Cox regression analysis. The prognostic risk score for each patient was calculated by the formula: Risk Score = ∑ (gene expression × coef). The patients were categorized into low- and high-risk groups based on the median risk score. Kaplan-Meier survival curves were used to examine the survival differences between the two groups. The effectiveness of the prognostic model was assessed using the receiver operating characteristic (ROC) curve and the area under the curve (AUC) calculation. The generalizability and robustness of the model were examined using two internal datasets, TCGA-test and TCGA-entire, and two external datasets, GSE41613 and GSE65858.

### 2.6 Relationship between Breg-related gene signature and clinicopathological characteristics

We utilized TCGA-HNSCC dataset and R packages such as ‘ggplot2’ and ‘ggpubr’ to examine the association between Breg-related gene signature and clinicopathological characteristics of patients with HNSCC. These tools were employed to visualize patterns and correlations within the data. Subgroup survival analysis of patients between different risk groups was performed using Kaplan-Meier survival curves.

### 2.7 Genomic variations and tumor mutation burden analysis

Mutation profiles and TMB were compared among different risk groups using the ‘maftools’ R package (23), in addition to ggplot2 and forestPlot (https://CRAN.R-project.org/package=forestplot). Specifically, somatic mutation data in Mutation Annotation Format were analyzed using the “maftools” R package (version 2.22.0). The functions read.maf, oncoplot, and mafCompare were applied using default parameters to visualize mutation landscapes and to identify differentially mutated genes between high- and low-risk groups. TMB was calculated as the total number of non-synonymous somatic mutations per megabase. Unless otherwise specified, all analyses were performed using default settings.

### 2.8 Construction of the nomogram based on the prognostic Breg-related gene signature

The risk score from the Breg-related gene signature was combined with various clinicopathological factors of the patients in TCGA-HNSCC dataset to construct a nomogram. The model was built using the ‘rms’ package in R (https://CRAN.R-project.org/package=rms). The predictive performance of the model for patient survival at 1-, 3-, and 5-years was evaluated using ROC curves to examine sensitivity and specificity. Calibration curves were used to assess the accuracy and clinical relevance of the model. The predictive performance was analyzed by calculating the concordance index (C-index) in conjunction with the AUC metrics. Kaplan-Meier survival curves were used to evaluate and compare survival differences across various risk categories in the nomogram model.

### 2.9 Weighted gene co-expression network analysis and GSEA

The ‘WGCNA’ R package (24) was used to construct a weighted gene co-expression network to identify gene modules that are associated with the Breg-related gene signature within the TCGA-HNSCC dataset. A soft threshold of 12 was applied to maintain a scale-free network architecture. Genes that demonstrated comparable expression profiles were classified into 12 distinct modules. The module with the strongest association with the Breg-related gene signature was selected for subsequent examination. The functional annotation and pathway enrichment analysis of this specific module were conducted using the R packages ‘clusterProfiler’, ‘org.Hs.eg.db’, and ‘enrichplot’ (https://bioconductor.org/packages/enrichplot/). GSEA was used to examine the differences in pathway enrichment between high- and low-risk groups. The ‘clusterProfiler’ R package was used for analyses to identify significantly enriched pathways with an adjusted *p*-value < 0.05.

### 2.10 Drug sensitivity analysis

The association between the Breg-related gene signature and the sensitivity to pharmacological agents was examined by using the NCI-60 cancer cell line panel. This panel included a wide range of cancer cell lines and response data for 792 chemotherapeutic and targeted therapies (25). Cell lines were assigned risk scores derived from the expression levels of eight signature genes and then classified into low- and high-risk categories. The Wilcoxon tests were used to compare the half maximal inhibitory concentration (IC_50_) values between the two groups, while Pearson correlation analysis was used to assess the correlation between risk scores and IC_50_ values. Visualizations were created with the ‘ggplot2’R package.

### 2.11 Cell culture

The HNSCC cell lines, notably SAS and SCC-9, were acquired from the Cell Bank of the Chinese Academy of Sciences, Shanghai, China. The SAS cell line was maintained in DMEM, whereas the SCC-9 cell line was cultivated in DMEM/F12 (Gibco, USA). Both culture media were enriched with 10% fetal bovine serum (FBS)(Gibco, USA) and antibiotics (100 U/mL penicillin and 0.1 mg/mL streptomycin), incubated at 37 °C in a humidified 5% CO_2_ environment.

### 2.12 OLR1 knockdown and real-time quantitative polymerase chain reaction assay

Small interfering RNAs (siRNAs) targeting the OLR1 gene were designed and acquired from GenePharma (Suzhou, China). The following siRNA sequences were used: si-NC sense: 5’-UUCUCCGAACGUGUCACGUTT-3’, si-NC antisense: 5’-ACGUGACACGUUCGGAGAATT-3’, si-OLR1–1 sense: 5’-CGGGCUCAUUUAACUGGGATT-3’, si-OLR1–1 antisense: 5’-UCCCAGUUAAAUGAGCCCGTT-3’, si-OLR1–2 sense: 5’- CUGUGCAUAUAUACAACGATT-3’, and si-OLR1–2 antisense: 5’-UCGUUGUAUAUAUGCACAGTT-3’. The transfection process adhered to the methodology described in our earlier study (26).

### 2.13 Cell proliferation and migration assays

The proliferative capacity of HNSCC cells after OLR1 knockdown of OLR1 was evaluated using the Cell Counting Kit-8 (CCK-8; Dojindo, Kumamoto, Japan) and the colony formation assay. For the CCK-8 assay, 2000 cells were plated per well in a 96-well format, incubated for 24 h, and then transfected with specific siRNAs. The optical density was calculated at 450 nm after 24, 48, and 72 h of transfection using a Sunrise microplate reader (Tecan, Switzerland). In the colony formation assay, 500 transfected cells were added to 6-well plates and allowed to grow for 12 days, with media replacements every four days. After incubation, the colonies were fixed in methanol for an hour and stained with Giemsa for 2 h at ambient temperature, and then manually counted. Images of the colonies were captured using an image scanner.

A wound healing assay, as previously described (25), was performed to evaluate the migration capacity of HNSCC cells after OLR1 knockdown. Cell migration was observed at 0, 6, and 9 h using an inverted microscope, and wound closure was quantitatively assessed with ImageJ software. Each experiment was conducted thrice.

### 2.14 Transcriptome sequencing following OLR1 gene knockdown

RNA sequencing (RNA-seq) was performed on the SAS cell line transfected with si-NC or si-OLR1 with five biological replicates per group to examine the molecular mechanisms underlying OLR1 knockdown in HNSCC. The RNA-seq library preparation and next-generation sequencing were performed using Personalbio (http://www.personalbio.cn/) in China. A gene was considered to be differentially expressed if its adjusted *p*-value was below 0.05 and absolute FC was greater than 1.5. The R packages tools ‘clusterProfiler’, ‘org.Hs.eg.db’, and ‘enrichplot’ were used for GO enrichment analysis. GSEA was conducted to identify enriched hallmark pathways between si-NC and si-OLR1 groups. Enrichment analyses were performed using the ‘clusterProfiler’ R package, considering pathways significantly enriched when the adjusted *p*-value was below 0.05.

### 2.15 Statistical analyses

Statistical analyses were conducted using R software (version 4.2.2). The Wilcoxon test or the two-tailed Student’s t-test was used for comparing two groups based on data distribution. The associations between variables were evaluated using Spearman correlation analysis. Kaplan-Meier survival curves were used to analyze the differences in OS among various groups, while the log-rank test was used to determine statistical significance. Cox regression analyses, both univariate and multivariate, were conducted to assess the prognostic significance of the Breg-related gene signature alongside clinicopathological factors. Statistical significance levels were defined as follows: *p < 0.05, **p < 0.01, ***p < 0.001, and ****p < 0.0001, with ‘ns’ denoting no significance.

## 3 Results

### 3.1 Breg-related genes define two immune subtypes of HNSCC with distinct microenvironmental features

**Figure 1:**
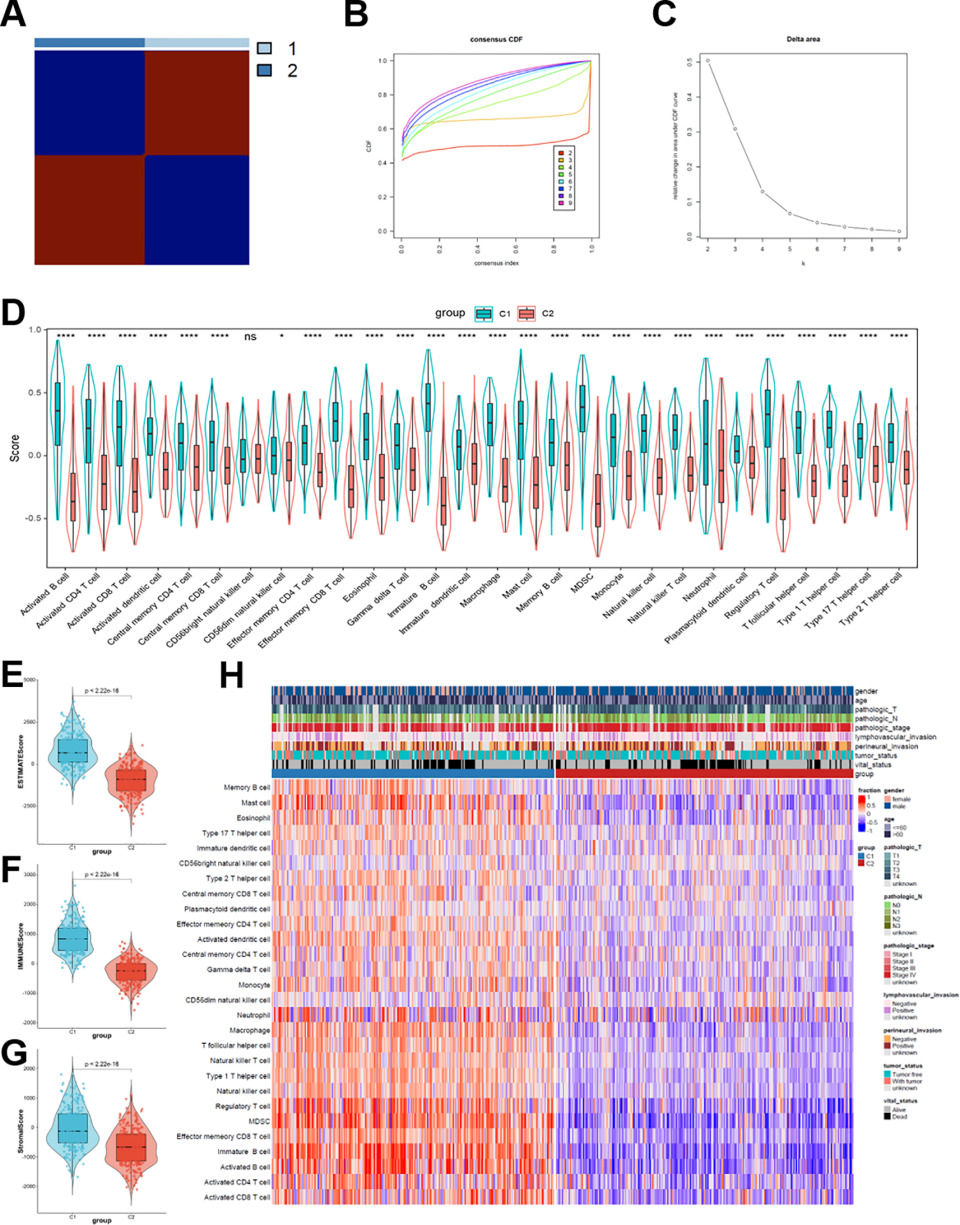
Breg-related genes-based molecular subtypes and their clinical relevance in the TCGA-HNSCC cohort. **(A)** Consensus clustering heatmap based on the expression profiles of Breg-related genes, identifying two distinct molecular subtypes (Clusters C1 and C2). **(B)** Cumulative distribution function (CDF) curves for different cluster numbers (k). **(C)** Relative change in the area under the CDF curve, identifying k = 2 as the optimal clustering solution. **(D)** Heatmap illustrating the expression patterns of Breg-related genes across clusters, annotated with inferred immune cell infiltration levels. **(E–G)** Comparison of ESTIMATE score, immune score, and stromal score between Cluster C1 and Cluster C2. **(H)** Heatmap displaying the relative abundance of 28 immune cell types estimated by ssGSEA across the two clusters, with color intensity indicating relative enrichment levels. Statistical significance between groups was assessed using the Wilcoxon test: *p < 0.05, **p < 0.01, ***p < 0.001, ****p < 0.0001, ns, not significant.

Consensus clustering analysis was conducted on the TCGA-HNSCC cohort. Breg-related genes were used to determine k = 2 to classify the cohort into two distinct molecular clusters, C1 and C2 (**Figures 1A–C**). The differential expression of multiple immunocytes in distinct clusters was compared, as illustrated in **Figure 1D**. The ESTIMATE algorithm was used to assess immune-related scores for characterizing the TME across both clusters. **Figures 1E–G** demonstrate that the ESTIMATE, immunological, and stromal scores were significantly higher in C1 than in C2 (all *p* < 0.05). A heatmap integrating 28 immune cell types with clinical characteristics of patients with HNSCC further highlighted the notable immunological heterogeneity among subtypes (**Figure 1H**). These results indicated that Breg-associated molecular subtypes captured distinct immunological landscapes of HNSCC, potentially accounting for differences in patient prognosis and therapeutic response.

### 3.2 Molecular heterogeneity and functional characterization of Breg-related subtypes in HNSCC

**Figure 2:**
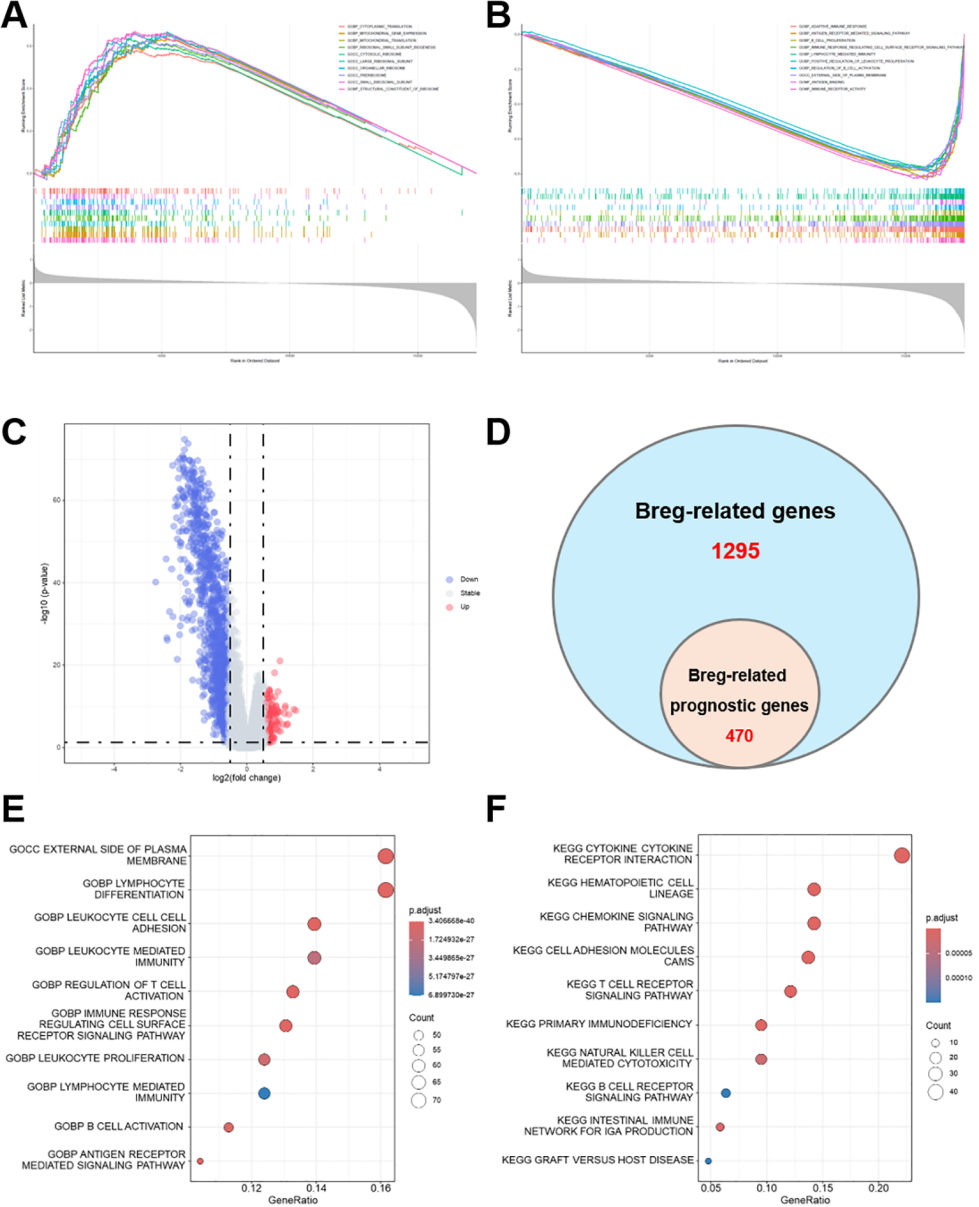
Differential gene expression and functional enrichment analyses between Breg-related molecular subtypes in the TCGA-HNSCC cohort. **(A, B)** GSEA plots comparing C2 versus C1, highlighting positively and negatively enriched biological pathways in C2 group. **(C)** Volcano plot illustrating DEGs between C1 and C2, with significantly upregulated and downregulated genes indicated. **(D)** Venn diagram demonstrating the intersection of Breg-related genes with prognostic genes identified using univariate Cox regression, yielding 470 Breg-related prognostic candidates. **(E)** GO enrichment analysis of DEGs, summarizing significantly overrepresented BP, cellular components (CC), and molecular functions (MF). **(F)** KEGG pathway enrichment analysis of DEGs, identifying critical signaling cascades potentially implicated in HNSCC pathogenesis.

C1 and C2 groups were compared using GSEA to assess the molecular characteristics linked to Breg-related subtypes in HNSCC. The C2 subtype exhibited significant positive enrichment of translation- and ribosome-associated pathways, including cytoplasmic translation, ribosomal subunit biogenesis, and structural constituents of the ribosome, as illustrated in **Figure 2A**. Mitochondrial gene expression and translation were notably enriched, indicating that C2 was characterized by increased protein synthesis capacity and metabolic activity. GSEA analysis indicated that the C2 subtype demonstrated significant negative enrichment in immune-related pathways, such as adaptive immune response, lymphocyte-mediated immunity, B cell proliferation and activation, antigen receptor-mediated signaling, and immune receptor activity (**Figure 2B**).

Differential expression analysis between C1 and C2 subtypes revealed a considerable number of significantly dysregulated genes, as illustrated in the volcano plot (**Figure 2C**). Univariate Cox regression analysis was conducted on the Breg-related gene sets to determine their prognostic significance. This analysis identified 470 prognostic Breg-related genes out of 1,295 candidates, highlighting their possible clinical significance (**Figure 2D**). GO enrichment analysis was used to assess the biological implications of these prognostic genes. The results revealed a significant enrichment in immune-related biological processes (BP) such as lymphocyte differentiation, leukocyte-mediated activation, T cell activation, B cell proliferation, and antigen receptor-mediated signaling (**Figure 2E**). KEGG pathway analysis revealed that the prognostic genes were primarily involved in immune regulatory signaling pathways, such as cytokine-cytokine receptor interaction, chemokine signaling, T/B cell receptor pathways, and natural killer cell-mediated cytotoxicity (**Figure 2F**). These results indicate that the transcriptional variations between C1 and C2 are closely associated with immune regulatory functions, with Breg-related prognostic genes possibly affecting the immune environment and clinical outcomes in HNSCC.

### 3.3 Development of a prognostic risk model based on Breg-related genes for patients with HNSCC

**Figure 3:**
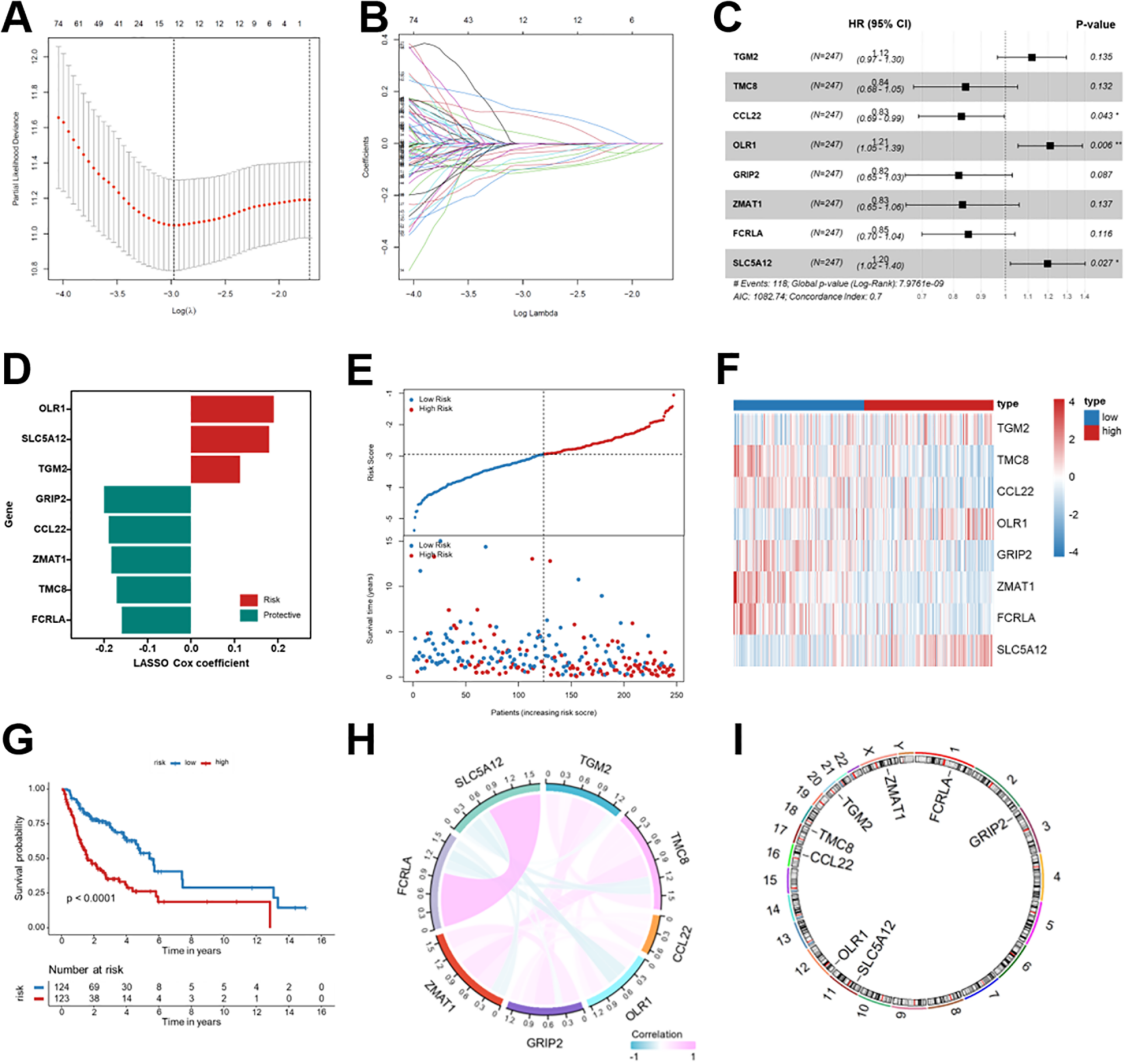
Construction of the Breg-related genes prognostic signature for patients with HNSCC. **(A, B)** LASSO Cox regression analysis for prognostic Breg-related genes in the TCGA-HNSCC training cohort. **(C)** Multivariate Cox regression analysis for the identified prognostic Breg-related genes. **(D)** Risk coefficients of the prognostic Breg-related genes. **(E)** Distribution of risk score and survival status of patients with HNSCC. **(F)** Expression profiles of the prognostic Breg-related genes between low- and high-risk groups. **(G)** Kaplan-Meier survival analysis for the OS of patients with HNSCC between low- and high-risk groups. **(H, I)** Correlation and chromosomal locations of the eight genes in the Breg-related gene signature. Statistical significance: *p < 0.05, **p < 0.01, ***p < 0.001, ****p < 0.0001, ns, not significant; HR, hazard ratios; CI, confidence intervals. High-risk patients represent those with elevated Breg-related risk scores, indicating a more immunosuppressive tumor microenvironment.

A univariate Cox regression analysis was performed on Breg-related genes using the TCGA-HNSCC training cohort, identifying 296 potential prognostic genes (**Supplementary Table 2**). LASSO regression was applied to these genes for dimensionality reduction and to prevent overfitting. As illustrated in **Figures 3A, B**, the optimal λ value was determined, and 12 genes (**Supplementary Table 3**) with non-zero coefficients were retained. To enhance the prognostic model, a stepwise multivariate Cox regression analysis was conducted, leading to the identification of eight genes (TGM2, TMC8, CCL22, OLR1, GRIP2, ZMAT1, FCRLA, SLC5A12) that were included in the prognostic signature, with the model achieving a C-index of 0.70 (**Figure 3C**). A risk score was computed for each patient using the coefficients from the prognostic signature. The formula used for calculating the risk score was: Riskscore = 0.19047 × OLR1 + 0.17950 × SLC5A12 + 0.11265 × TGM2 + (-0.19971) × GRIP2 + (-0.18878) × CCL22 + (-0.18352) × ZMAT1 + (-0.17045) × TMC8 + (-0.15858) × FCRLA (**Figure 3D**). According to the analysis of risk scores and survival status, the high-risk group demonstrated a greater mortality rate than the low-risk group (**Figure 3E**). Expression profiling revealed distinct expression patterns of the eight genes in the two risk groups (**Figure 3F**). To further quantitatively validate the consistency of gene expression patterns underlying the risk stratification, we systematically compared the expression levels of the eight signature genes between high- and low-risk groups across the TCGA training cohort, TCGA validation cohorts, and external GEO datasets. The results demonstrated highly consistent expression trends across cohorts, which were concordant with the regression coefficients of the prognostic model (**Supplementary Table 4**). Kaplan-Meier survival analysis demonstrated considerably worse OS in the high-risk group than the low-risk group (*p* < 0.001, **Figure 3G**). Lastly, the correlation and chromosomal locations of the eight prognostic genes were mapped in **Figures 3H, I**. These results imply that the Breg-related prognostic signature successfully classified the patients with HNSCC into risk groups with significantly varied survival outcomes.

### 3.4 Validation of the Breg-related prognostic signature in independent HNSCC cohorts

**Figure 4:**
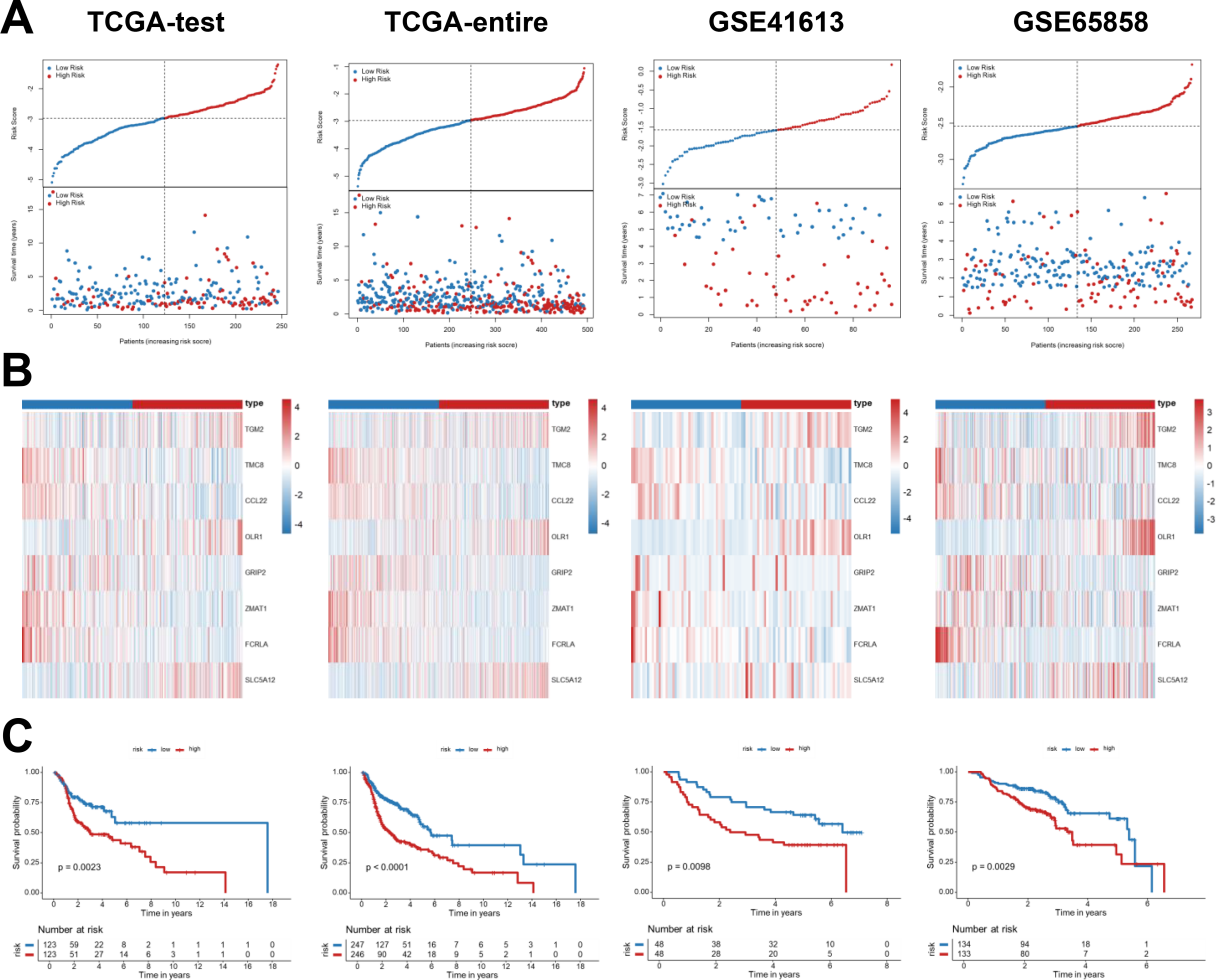
Validation of the prognostic Breg-related gene signature. **(A)** Distribution of risk score and survival status of patients with HNSCC in several validation cohorts, including TCGA-test, TCGA-entire, GSE41613, and GSE65858 datasets. **(B)** Expression profiles of the prognostic Breg-related genes between low- and high-risk groups in the validation cohorts. **(C)** Kaplan-Meier survival analysis for the OS of patients with HNSCC between low- and high-risk groups among validation cohorts. High-risk patients represent those with elevated Breg-related risk scores, indicating a more immunosuppressive tumor microenvironment.

The Breg-related gene signature was verified in several independent cohorts, including TCGA-test, TCGA-entire, GSE41613, and GSE65858 datasets, to assess the robustness and generalizability of the prognostic signature. High-risk patients continuously exhibited higher mortality rates than those of the low-risk group in all validation cohorts (**Figure 4A**). Expression heatmaps revealed distinct expression patterns of the prognostic model between low- and high-risk groups, highlighting the discriminative power of the signature (**Figure 4B**). The Kaplan-Meier survival analysis demonstrated that patients categorized as high-risk had significantly lower OS rates than their low-risk counterparts across all cohorts (*p* < 0.05, **Figure 4C**). The Breg-related prognostic signature consistently predicts survival outcomes for patients with HNSCC across many datasets.

### 3.5 Association between Breg-related prognostic signature and clinicopathological characteristics in patients with HNSCC

**Figure 5:**
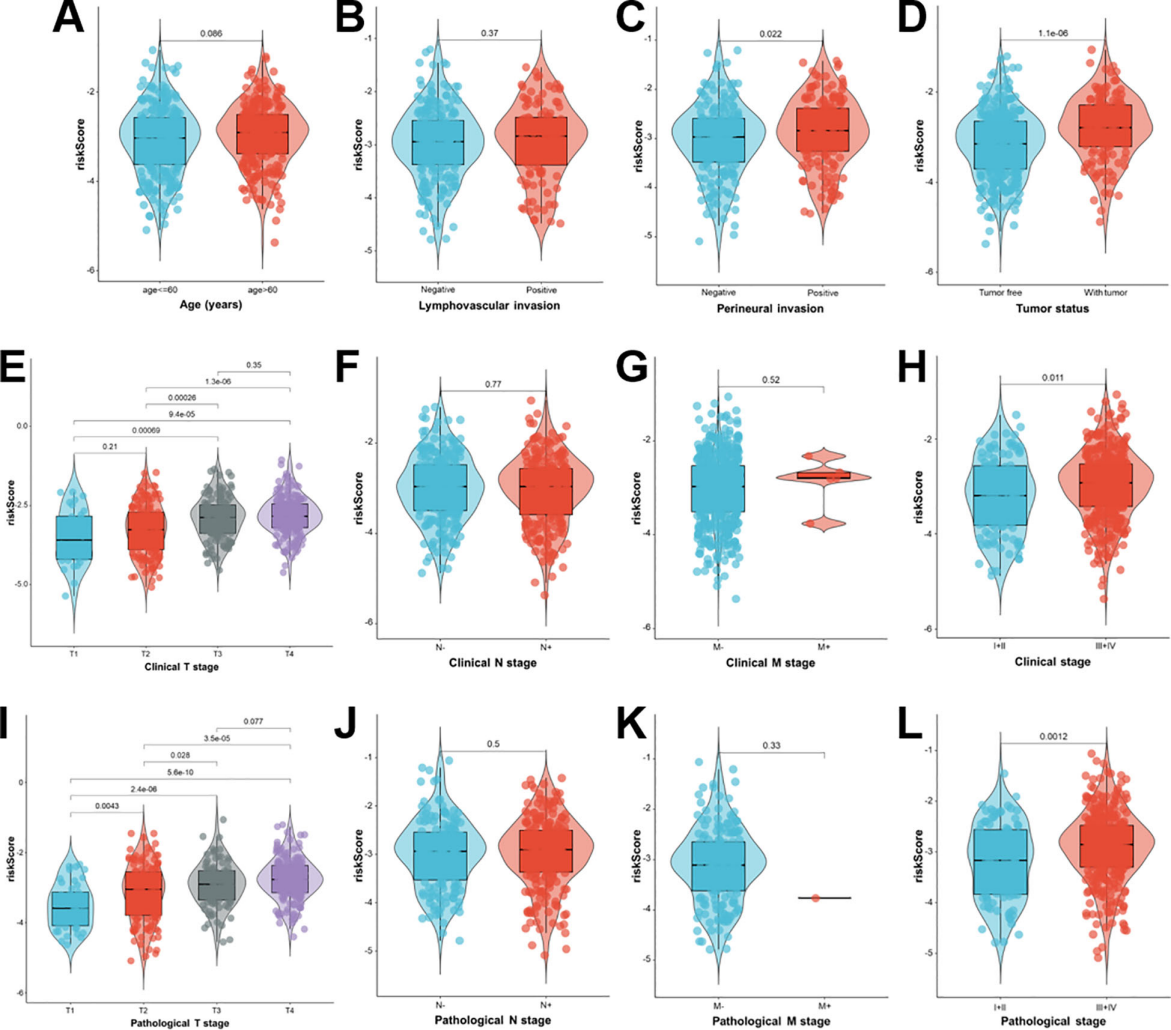
Association of Breg-related gene signature-derived risk scores with clinicopathological characteristics in the TCGA-HNSCC cohort. Violin plots illustrate the distribution of risk scores across subgroups stratified by multiple clinicopathological characteristics in patients with HNSCC. Statistical significance between groups was assessed using the Wilcoxon test.

The association between risk scores and other clinicopathological features in the TCGA-HNSCC cohort was evaluated to assess the clinical importance of the Breg-related prognostic signature. This study analyzed clinical characteristics such as age, lymphovascular and perineural invasion, tumor status, clinical and pathological T/N/M stages, and overall clinical and pathological stages (**Figures 5A–L**). The findings revealed that higher risk scores were markedly correlated with perineural invasion, tumor status, advanced clinical T stage, higher overall clinical stage, advanced pathological T stage, and higher overall pathological stage (all *p* < 0.05). The Breg-related gene signature risk score is strongly associated with aggressive clinicopathological features, suggesting its potential use in detecting malignant progression in patients with HNSCC.

### 3.6 Subgroup survival analyses of patients with HNSCC stratified by the Breg-related gene signature

**Figure 6:**
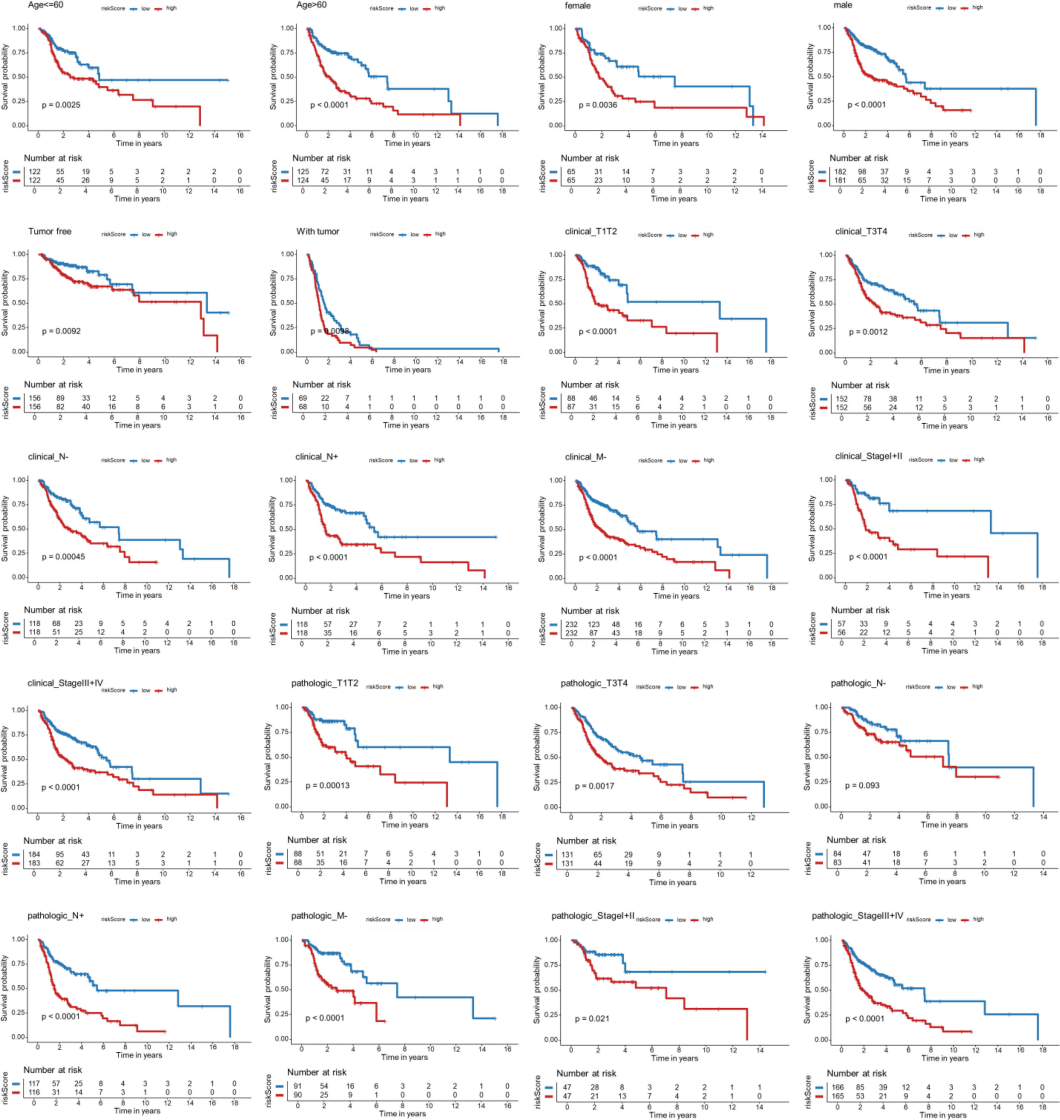
Kaplan-Meier subgroup survival analyses of TCGA-HNSCC patients stratified by the Breg-related gene signature. **(A)** Kaplan-Meier survival curves comparing OS between high- and low-risk groups across different subgroups. High-risk patients represent those with elevated Breg-related risk scores, indicating a more immunosuppressive tumor microenvironment.

Kaplan-Meier subgroup survival analyses were conducted in the TCGA-HNSCC cohort to further evaluate the prognostic usefulness of the Breg-related gene signature across various clinical backgrounds (**Figure 6**). High-risk patients exhibited significantly worse OS across almost all subgroups when stratified by age, sex, tumor status, clinical T/N/M stage, overall clinical stage, pathological T/N/M stage, and overall pathological stage. These findings verified that the Breg-related gene signature offers robust prognostic stratification for patients with HNSCC in a variety of clinicopathological contexts.

### 3.7 Genomic alterations and TMB associated with the Breg-related prognostic signature in HNSCC

**Figure 7:**
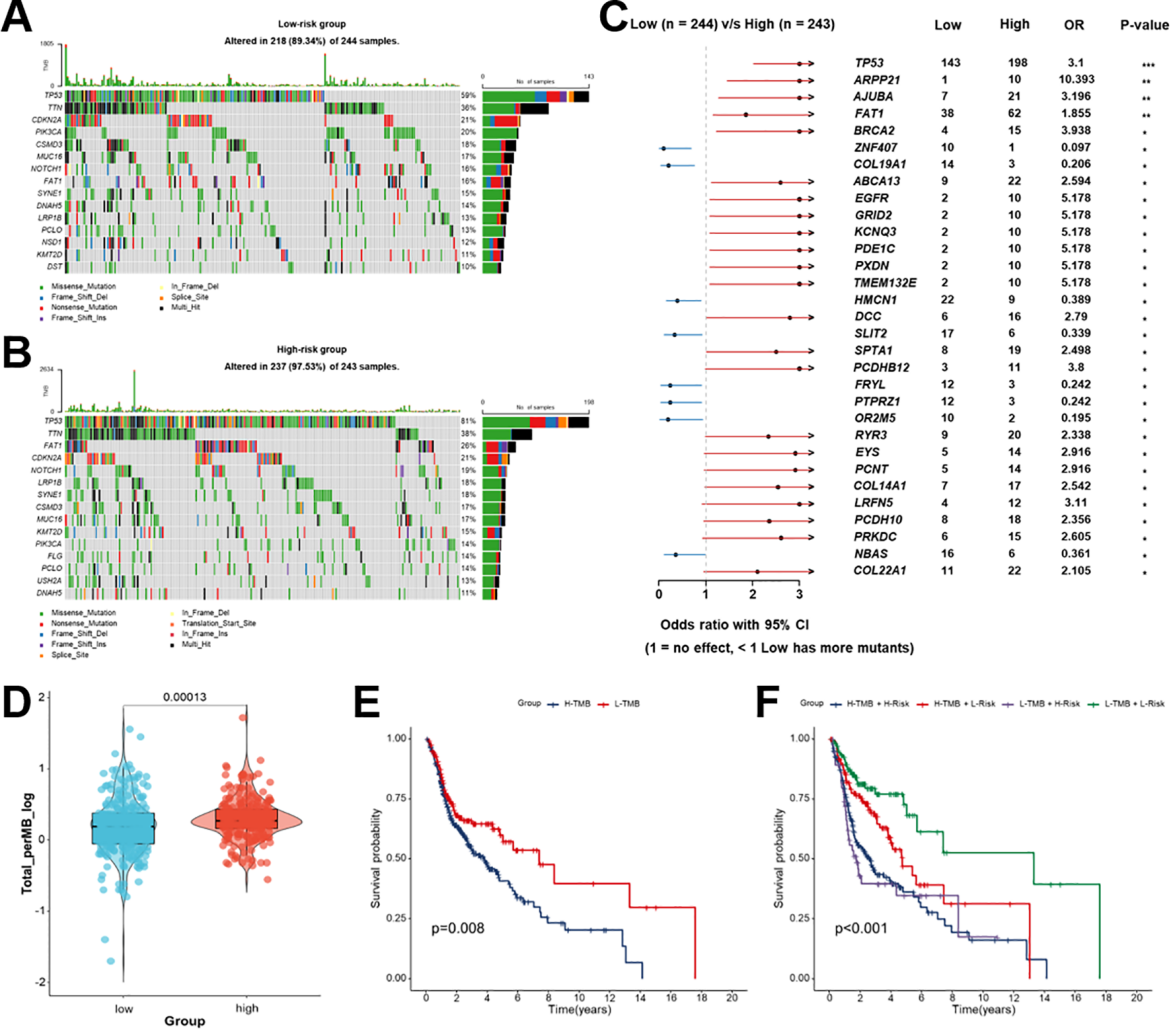
Analysis of genomic alterations and tumor mutation burden (TMB) in low- and high-risk patients with HNSCC. **(A, B)** Mutational landscape of the top mutated genes in the low- **(A)** and high- **(B)** risk groups based on the Breg-related gene signature from TCGA-HNSCC patients. Mutation types are color-coded, and mutation frequencies are displayed on the right. **(C)** Forest plot comparing significantly mutated genes between low- and high-risk groups, with odds ratio and 95% CI. **(D)** Comparing TMB levels between low- and high-risk groups. **(E)** Kaplan-Meier survival curves for patients with HNSCC stratified by TMB status. **(F)** Kaplan-Meier survival analysis of four subgroups defined by both risk score and TMB status. Statistical significance between groups was assessed using the Wilcoxon test: *p < 0.05, **p < 0.01, ***p < 0.001, ****p < 0.0001, ns, not significant. High-risk patients represent those with elevated Breg-related risk scores, indicating a more immunosuppressive tumor microenvironment.

The mutational landscape of TCGA-HNSCC patients demonstrated a marked difference between the two risk groups. Common driver mutations such as TP53, TTN, FAT1, and CDKN2A were commonly found in low- and high-risk groups observed, but their frequency differed substantially (**Figures 7A, B**). A comparison of the mutational spectra of the two groups highlighted a number of genes with notably differing mutation rates. Some gene mutations, such as TP53, FAT1, BRCA2, and EGFR, were more common in high-risk patients, while other alterations, such as ZNF407, COL19A1, and HMCN1, were enriched in the low-risk group, as indicated by the forest plot (**Figure 7C**). This suggests distinct genomic backgrounds underlying prognostic stratification.

Additionally, this study assessed the correlation between risk group and TMB in patients with HNSCC. High-risk patients demonstrated significantly higher TMB (*p* = 0.00013, **Figure 7D**) and worse OS (*p* = 0.008, **Figure 7E**) than the low-risk group. Patients with low-risk scores and low TMB had the best outcomes, whereas those with high-risk scores and high TMB had the worst survival (*p* < 0.001, **Figure 7F**). This was evident through refined risk stratification enabled by integrating TMB status with the Breg-related signature. Collectively, these findings demonstrated that the Breg-related gene signature reflects underlying genomic alterations and TMB levels, in addition to reinforcing its prognostic utility in HNSCC.

### 3.8 Nomogram based on Breg-related gene signature predicts survival in HNSCC

**Figure 8:**
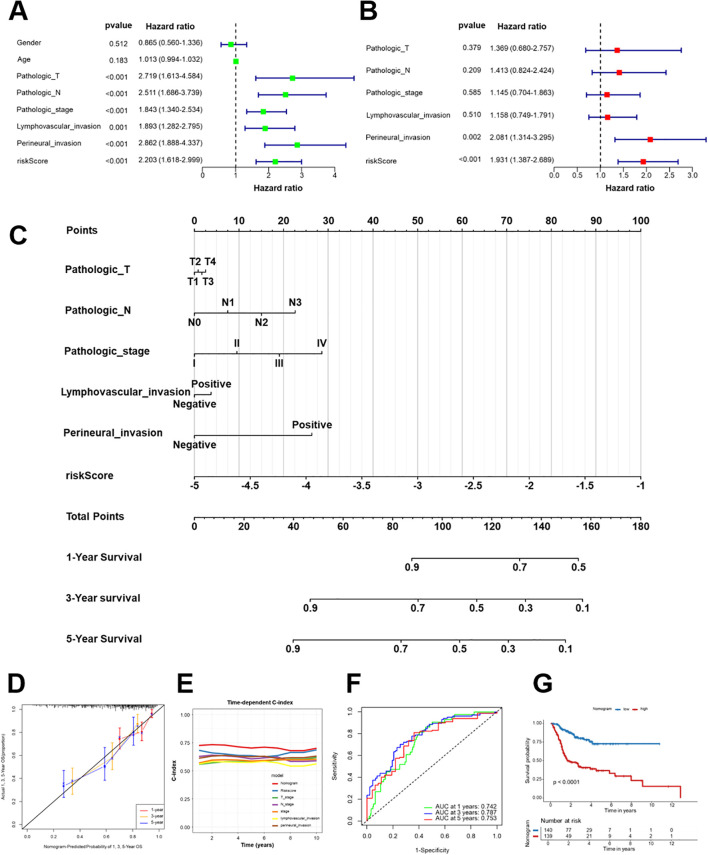
A nomogram developed from the Breg-related gene signature to predict the OS of patients with HNSCC. **(A, B)** Univariate **(A)** and multivariate **(B)** Cox regression analyses evaluating the prognostic significance of the risk score and key clinicopathological features of TCGA-HNSCC patients. **(C)** Nomogram incorporating significant prognostic factors for predicting 1-, 3-, and 5-year OS in patients with HNSCC. **(D)** Calibration curves demonstrate the agreement between predicted and observed survival probabilities at 1-, 3-, and 5-year intervals. **(E)** Time-dependent C-index for the nomogram and individual prognostic factors. **(F)** ROC curves demonstrate the predictive performance of the nomogram at 1-, 3-, and 5-year points. **(G)** Kaplan-Meier survival analysis stratifying patients into low- and high-risk groups according to the nomogram score, with survival differences assessed by the log-rank test.

According to univariate and multivariate Cox regression analyses, the risk score derived from the Breg-related gene signature independently predicts OS in TCGA-HNSCC patients (**Figures 8A, B**). A nomogram was developed based on the important predictors to assess the 1-, 3-, and 5-year OS probability for patients with HNSCC (**Figure 8C**). The reliability of the nomogram was verified by the calibration curves, which demonstrated strong alignment between predicted and actual outcomes (**Figure 8D**). The nomogram consistently outperformed individual clinical parameters in predicting patient prognosis, as evidenced by the time-dependent C-index (**Figure 8E**). ROC curve analysis validated the predictive performance of the nomogram, with AUC values of 0.72, 0.75, and 0.73 for 1-, 3-, and 5-year survival, respectively (**Figure 8F**). Kaplan-Meier survival analysis revealed significantly different survival outcomes between high-risk and low-risk groups, as determined by the nomogram score, with the high-risk group exhibiting a considerably worse prognosis (*p* < 0.001, **Figure 8G**). These results indicated that the nomogram serves as an effective tool for personalized survival prediction in HNSCC as it integrates the Breg-related gene signature with clinicopathological factors.

### 3.9 WGCNA identifies prognostic modules and immune-related pathways in patients with HNSCC

**Figure 9:**
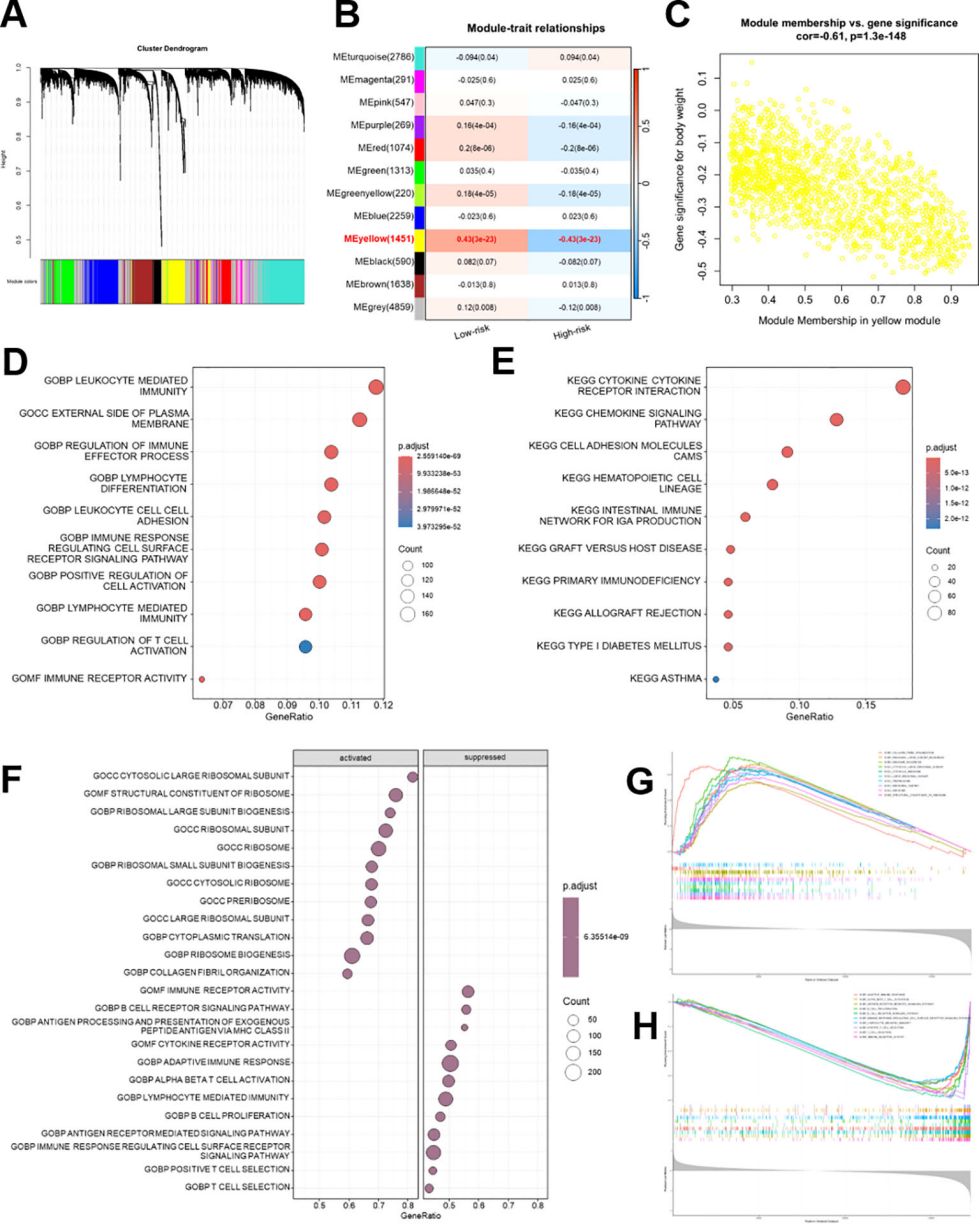
WGCNA for the Breg-related gene signature in patients with HNSCC. **(A)** Hierarchical clustering dendrogram of genes with different colors representing distinct co-expression modules identified by WGCNA in the TCGA-HNASCC cohort. **(B)** The heatmap demonstrates the correlation between each gene module and risk groups. **(C)** Scatter plot of module membership versus gene significance for the yellow module, revealing a strong positive correlation between module membership and prognostic relevance. **(D, E)** GO **(D)** and KEGG **(E)** enrichment analyses of genes in the yellow module. **(F)** GSEA analysis (GO terms) comparing activated and suppressed BP between high- and low-risk groups. **(G, H)** Enrichment plots of key hallmark pathways activated **(G)** and suppressed **(H)** in the high-risk group.

WGCNA was used to develop a gene co-expression network from TCGA-HNSCC data, identifying multiple distinct modules, each represented by a unique color (**Figure 9A**). The yellow module demonstrated the strongest correlation with the risk groups, having a correlation coefficient of -0.43 and a *p*-value of 3e-23 (**Figure 9B**). A significant positive association was observed between the gene significance and yellow module membership (correlation = 0.61, *p* = 1.3e-148), underscoring the crucial function of this module in prognosis (**Figure 9C**). Functional enrichment analysis of the yellow module genes identified a significant enrichment of GO terms linked to immune-related processes, such as lymphocyte-mediated immunity, regulation of immune effector processes, and immune receptor activity (**Figure 9D**). KEGG analysis consistently revealed a significant enrichment in immune-related pathways, such as cytokine-cytokine receptor interaction, chemokine signaling, and cell adhesion molecules (**Figure 9E**).

GSEA analysis of GO terms demonstrated activation of ribosome-associated pathways and suppression of immune receptor signaling pathways in high-risk groups (**Figure 9F**). The high-risk group exhibited positive enrichment in pathways primarily associated with ribosome biogenesis and protein synthesis (**Figure 9G**). In contrast, negatively enriched pathways were mainly linked to immune activity (**Figure 9H**).

### 3.10 Immune infiltration patterns associated with the Breg-related gene signature

**Figure 10:**
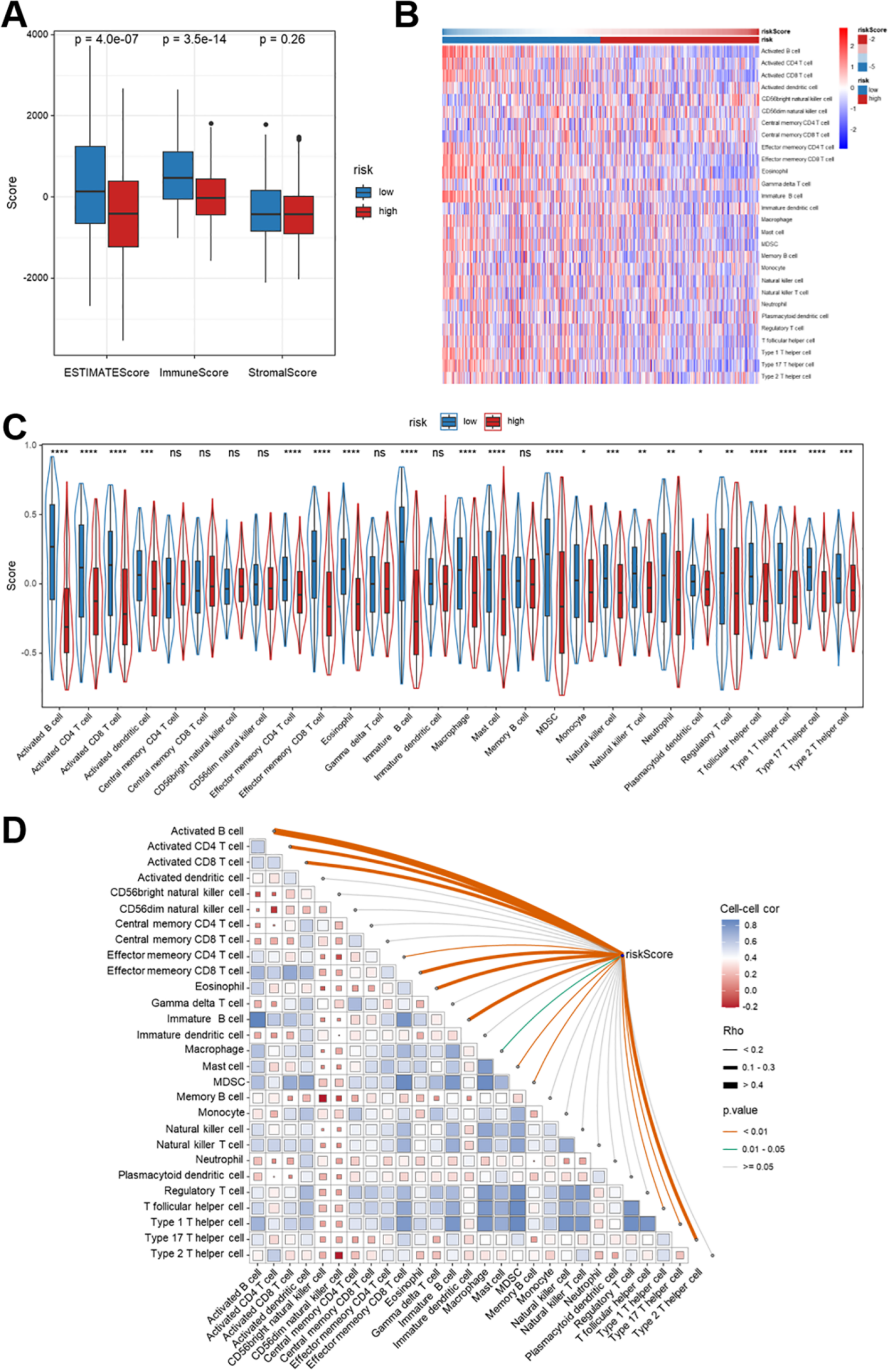
Immune infiltration characteristics associated with the Breg-related gene signature in TCGA-HNSCC patients. **(A)** Comparison of ESTIMATE, immunological, and stromal scores between low- and high-risk groups as defined by Breg-related gene signature-derived risk score. **(B)** Heatmap showing the relative infiltration levels of 28 immune cell subpopulations estimated by ssGSEA; color gradients indicate relative enrichment (red, high; blue, low). **(C)** Violin plots illustrating differences in immune cell infiltration between low- and high-risk groups. **(D)** Spearman correlation analysis between the Breg-related risk score and immune cell infiltration. Square colors indicate the direction and strength of correlations among immune cell types, while curved lines illustrate correlations between immune cell subsets and the risk score, with line thickness and color reflecting correlation magnitude and statistical significance. Statistical significance between groups was assessed using the Wilcoxon test: *p < 0.05, **p < 0.01, ***p < 0.001, ****p < 0.0001, ns, not significant. High-risk patients represent those with elevated Breg-related risk scores, indicating a more immunosuppressive tumor microenvironment.

Immune infiltration analysis using the ESTIMATE algorithm demonstrates distinct variations between risk groups based on the Breg-related gene signature. High-risk patients exhibited substantially lower ESTIMATE score (*p* = 4.0e-07) and immune score (*p* = 3.5e-14), while the stromal score did not differ significantly (*p* = 0.26) (**Figure 10A**). **Figure 10B** illustrates a heatmap revealing the distribution of 28 immune cell subpopulations across low- and high-risk groups. In contrast, **Figure 10C** displays a violin plot that compares the risk scores between these groups. Correlation matrix analysis further verified these findings, suggesting a negative association between risk score and effector immune cell subsets (**Figure 10D**). This study used various immune cell deconvolution algorithms, such as XCell, TIMER, QUANTISEQ, MCPCOUNTER, CIBERSORT-ABS, CIBERSORT, to verify changes in the immune landscape. The results consistently demonstrated that high-risk patients had fewer anti-tumor immune cells and increased immunosuppressive cell infiltration, thereby supporting the idea of an immunosuppressive TME (**Supplementary Figure 1**). All these results collectively revealed that the Breg-related gene signature delineates immune infiltration patterns in the TME of HNSCC.

### 3.11 Association of risk score with immune checkpoint expression and immunotherapy response

**Figure 11:**
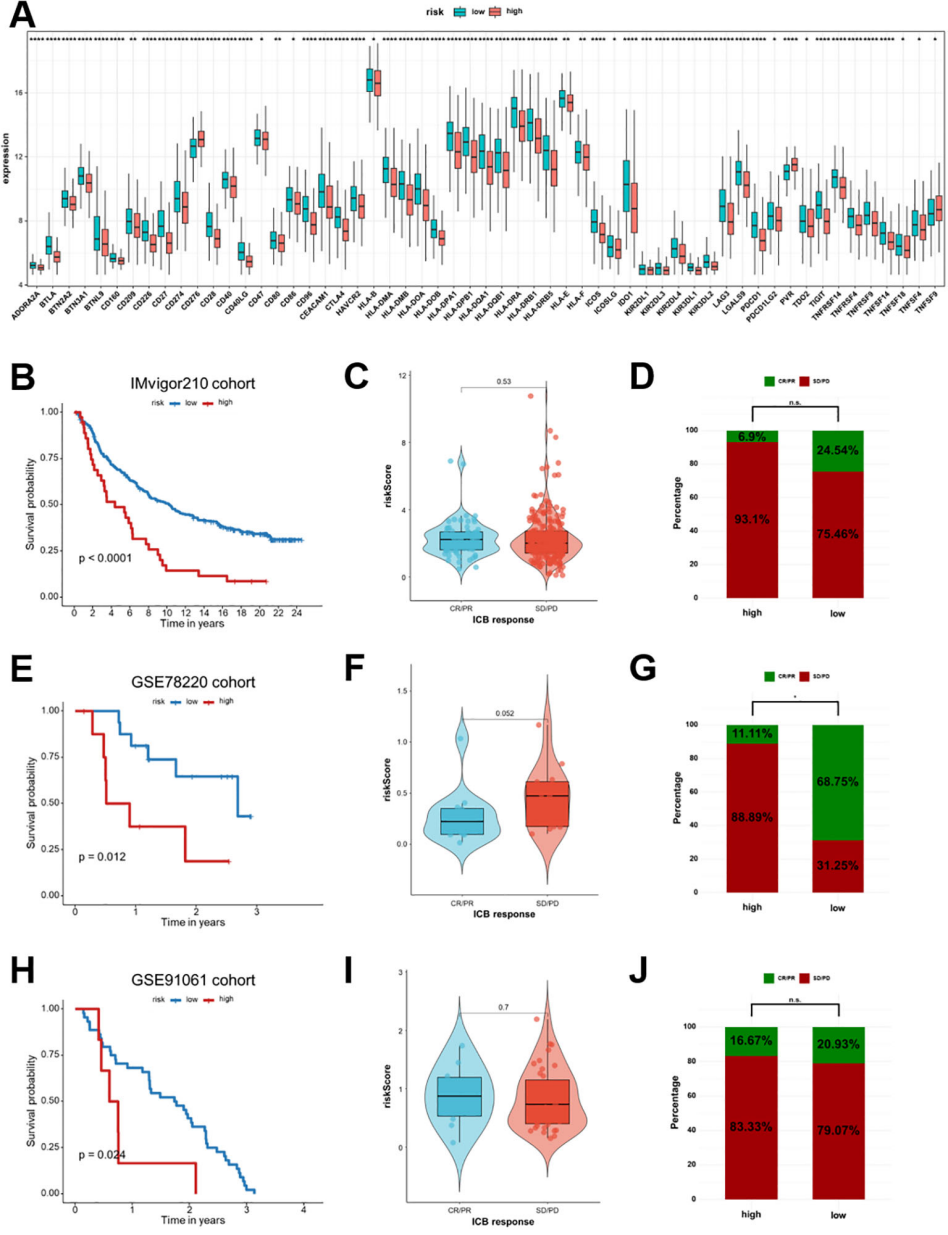
Association of the Breg-related gene signature with immune checkpoint expression and immunotherapy response in independent cohorts. **(A)** Boxplots comparing the expression levels of multiple immune checkpoint molecules between low- and high-risk groups in the TCGA-HNSCC cohort. **(B, E, H)** Survival analysis for IMvigor210, GSE78220, and GSE91061 immunotherapy cohorts, respectively. **(C, F, I)** Distribution of risk scores between CR/PR and SD/PD disease groups after patients received ICB therapy in the above cohorts. **(D, G, J)** Proportion of CR/PR and SD/PD cases in low- and high-risk groups for the above cohorts. Statistical significance between groups was assessed using the Wilcoxon test: *p < 0.05, **p < 0.01, ***p < 0.001, ****p < 0.0001, ns, not significant. High-risk patients represent those with elevated Breg-related risk scores, indicating a more immunosuppressive tumor microenvironment.

It was also investigated whether the risk score can represent differences in checkpoint expression and predict response to immune checkpoint blockade (ICB) therapy. Low-risk patients revealed significantly elevated expression of immune checkpoint molecules such as PDCD1 (PD-1), CD274 (PD-L1), and CTLA-4 in the TCGA-HNSCC cohort, indicating a more active TME (**Figure 11A**). High-resolution versions of the plots showing checkpoint molecule expression are provided in the **Supplementary Materials**. The low-risk group consistently outperformed the high-risk group in three independent immunotherapy cohorts concerning survival outcomes (IMvigor210, *p* < 0.0001; GSE78220, *p* = 0.012; GSE91061, *p* = 0.024; **Figures 11B, E, H**). Risk scores were also associated with clinical responses to ICB therapy. Patients achieving complete or partial responses (CR/PR) typically had lower risk scores, while those with stable or progressive disease (SD/PD) exhibited higher scores (**Figures 11C, F, I**). The low-risk group consistently exhibited a higher proportion of CR/PR cases, even though statistical significance was not achieved in specific cohorts (IMvigor210: 24.54% versus 6.9%; GSE78220: 68.75% versus 11.11%; GSE91061: 20.93% versus 16.67%; **Figures 11D, G, J**). These results highlight that the risk score can predict potential benefits from ICB therapy, given that low-risk patients have improved survival and a greater likelihood of treatment response.

### 3.12 Association between Breg-related risk score and drug sensitivity

**Figure 12:**
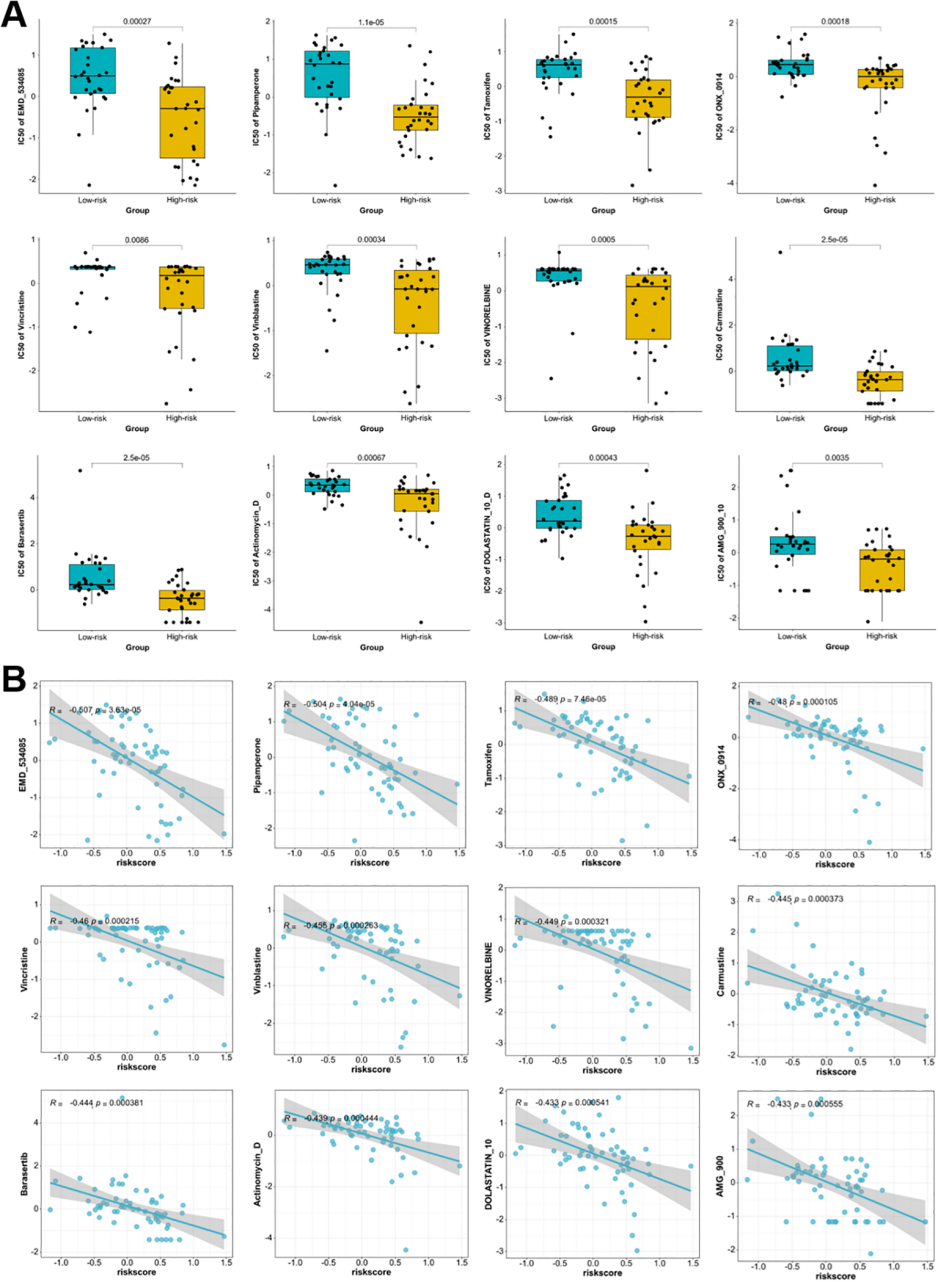
Association between the Breg-related gene signature and drug sensitivity in TCGA-HNSCC patients. **(A)** Comparison of the estimated half-maximal IC_50_ values for multiple anticancer agents between low- and high-risk groups as defined by the nomogram-derived risk score. **(B)** Spearman correlation analysis between risk score and drug sensitivity for multiple sets of agents. Statistical significance between groups was assessed using the Wilcoxon test. High-risk patients represent those with elevated Breg-related risk scores, indicating a more immunosuppressive tumor microenvironment.

This study also analyzed the variations in drug sensitivity between the low-risk and high-risk groups to evaluate the clinical relevance of the Breg-related risk model in guiding treatment strategies. **Figure 12A** illustrates that high-risk group exhibited significantly lower IC_50_ values for chemotherapy and targeted agents such as Vincristine, Actinomycin D, Tamoxifen, and Barasertib (all *p* < 0.01), indicating increased sensitivity and potential benefit from these treatments. Correlation analyses supported these findings, suggesting significant negative associations between risk score and drug response for various agents (**Figure 12B**). For instance, strong inverse correlations were observed for Vincristine (R = -0.460, *p* < 0.0001), Actinomycin D (R = -0.439, *p* < 0.0001), Tamoxifen (R = -0.489, *p* < 0.0001), and Barasertib (R = -0.444, *p* < 0.0001). Full-resolution figures illustrating drug sensitivity analyses are included in the **Supplementary Materials**. These findings suggest that the Breg-related risk signature reflects differential drug response profiles, which may provide valuable guidance for individualized treatment strategies in HNSCC.

### 3.13 OLR1 as a key gene with pan-cancer expression and prognostic value

**Figure 13:**
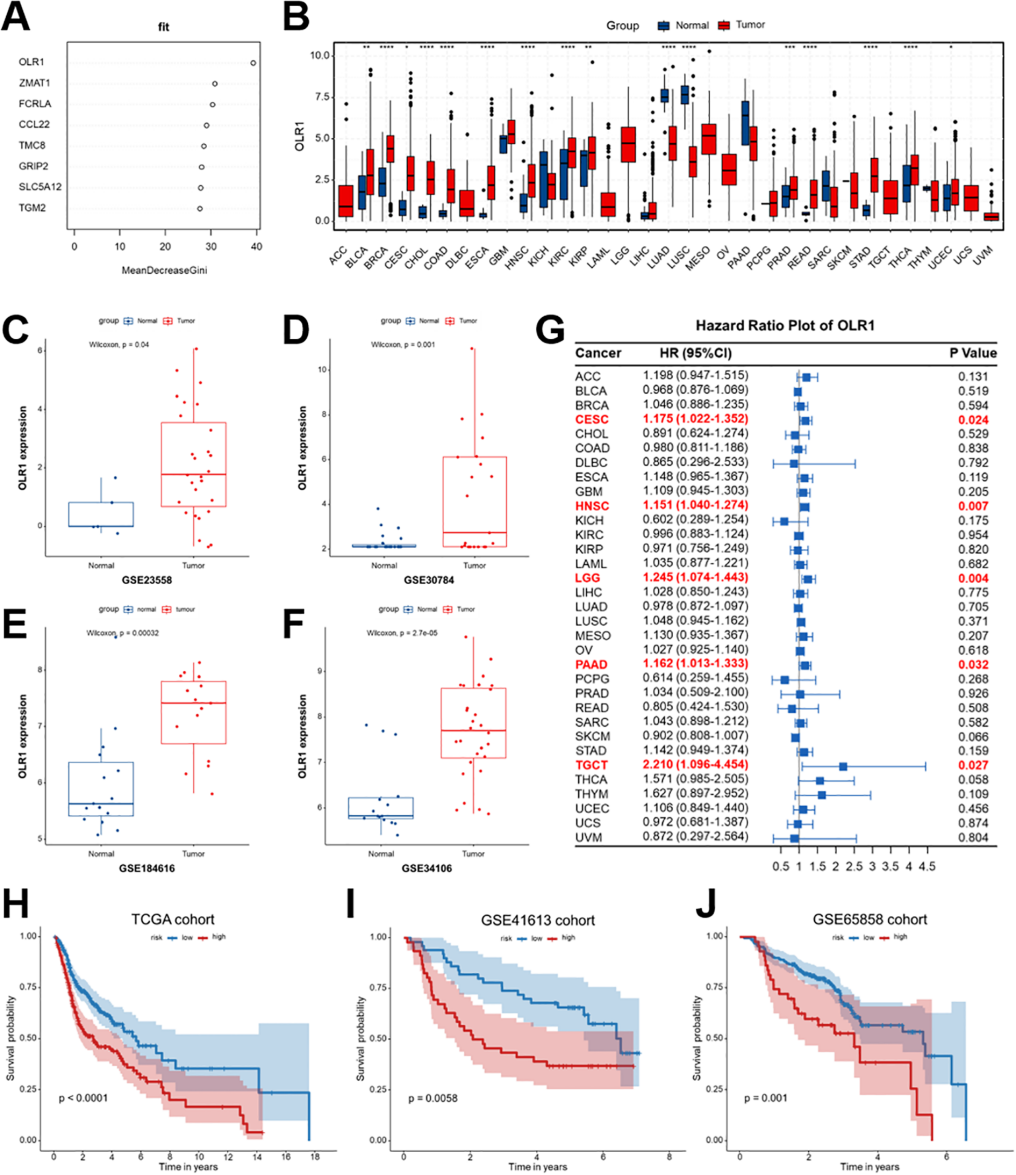
Identification of OLR1 as a key gene and its pan-cancer expression and prognostic significance. **(A)** Random forest analysis ranking the significance of candidate genes, with OLR1 identified as the top feature based on mean decrease in Gini index. **(B)** Pan-cancer comparison of OLR1 expression between tumor and normal tissues across multiple TCGA cancer types. **(C–F)** Validation of OLR1 expression differences between tumor and normal tissues in independent GEO datasets (GSE23558, GSE30784, GSE146416, and GSE34106). **(G)** Forest plot summarizing hazard ratio and 95% confidence interval (95%CI) for OLR1 expression in relation to OS across various cancer types, with significant associations highlighted in red. **(H–J)** Kaplan-Meier survival analyses revealing the prognostic value of OLR1 expression in the TCGA-HNSCC cohort **(H)** and independent validation cohorts GSE41613 **(I)** and GSE65858 **(J)**. Statistical significance between groups was assessed using the Wilcoxon test: *p < 0.05, **p < 0.01, ***p < 0.001, ****p < 0.0001, ns, not significant.

Random forest analysis identified OLR1 as the top-ranked gene among the candidate features, indicating its crucial role in the Breg-related signature (**Figure 13A**). OLR1 expression was markedly elevated in a variety of tumor tissues in the TCGA pan-cancer cohort compared with matched normal counterparts (**Figure 13B**). Analyses of various independent GEO datasets revealed that OLR1 expression was significantly higher in HNSCC tumor tissues than in adjacent normal tissues (**Figures 13C–F**). Survival analyses indicated that higher OLR1 expression was associated with poorer OS in several cancer types, most notably HNSCC (**Figure 13G**). Kaplan-Meier curves revealed that the patients with HNSCC with higher OLR1 expression had significantly lower survival in TCGA and external validation cohorts (GSE41613 and GSE65858) (**Figures 13H–J**). These results collectively suggest that OLR1 functions as a key oncogenic gene and represents a robust prognostic biomarker in HNSCC.

### 3.14 OLR1 knockdown inhibits the proliferation and migration of HNSCC cells

**Figure 14:**
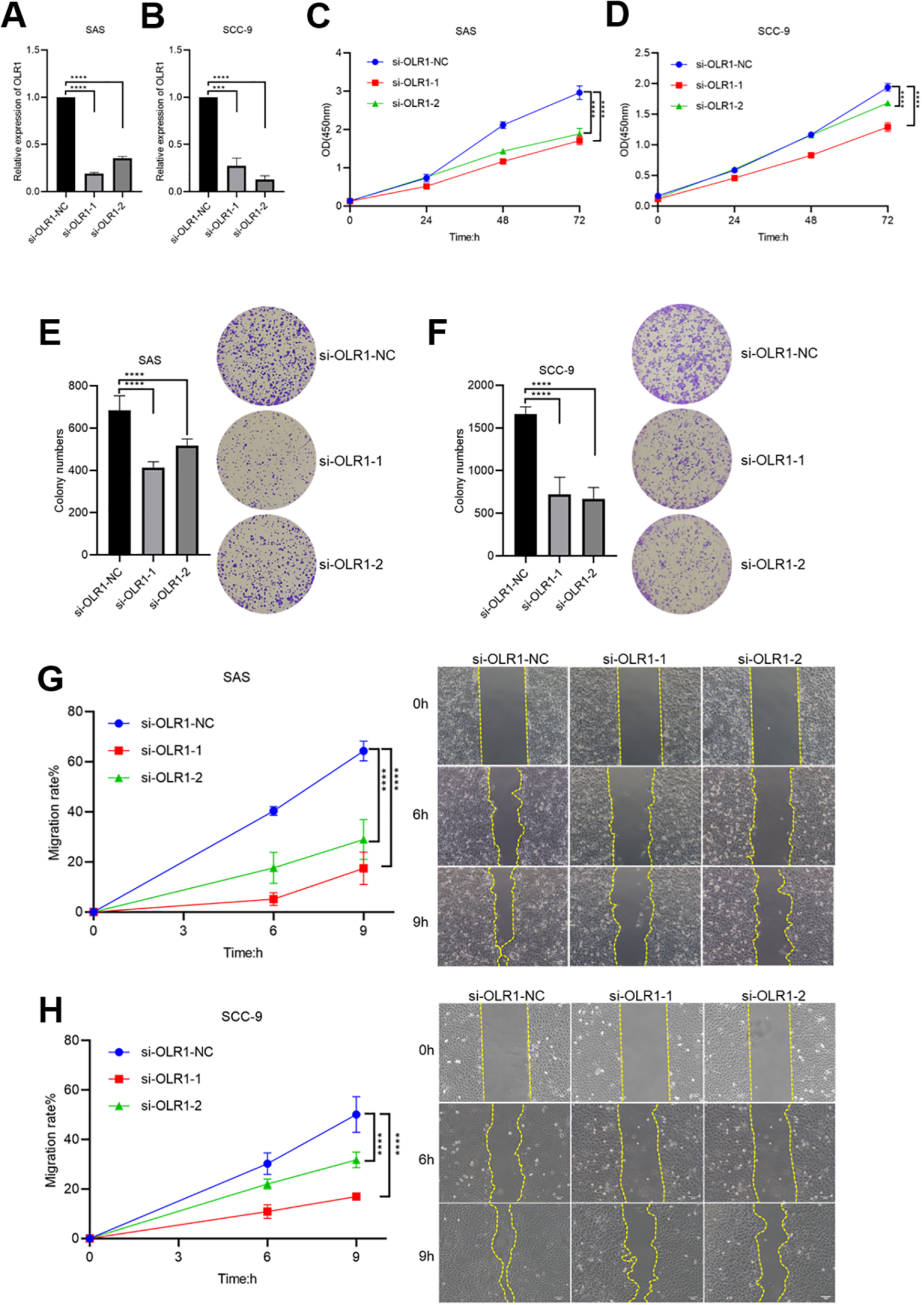
Effects of OLR1 knockdown on proliferation and migration in HNSCC cell lines. **(A, B)** QRT-PCR analysis confirming the knockdown efficiency of OLR1 in SAS **(A)** and SCC-9 **(B)** cells. **(C, D)** Cell proliferation assessed by CCK-8 assay at 0, 24, 48, and 72 h after OLR1 knockdown in SAS **(C)** and SCC-9 **(D)** cells. **(E, F)** Representative images and quantification of colony formation assays in SAS **(E)** and SCC-9 **(F)** cells after OLR1 knockdown. **(G, H)** Wound healing assays demonstrating the migration rates in SAS **(G)** and SCC-9 **(H)** cells after OLR1 knockdown. Statistical significance: *p < 0.05, **p < 0.01, ***p < 0.001, ****p < 0.0001, ns, not significant.

Functional experiments using SAS and SCC-9 cell lines were conducted in this study to better understand the biological role of OLR1 in HNSCC. For instance, qRT-PCR analysis revealed a significant decrease in OLR1 expression using two independent siRNAs (si-OLR1–1 and si-OLR1-2) compared to the negative control (si-OLR1-NC) (**Figures 14A, B**). CCK-8 assays demonstrated that OLR1 silencing significantly inhibited cell proliferation in SAS and SCC-9 cells over time (**Figures 14C, D**). Similarly, colony formation assays indicated that OLR1 knockdown significantly reduced the number of colonies in both cell lines, indicating impaired clonogenic potential (**Figures 14E, F**). Wound healing assays demonstrated that silencing OLR1 significantly impaired the migratory ability of SAS and SCC-9 cells, as indicated by decreased wound closure rates (**Figures 14G, H**). These findings collectively suggest that OLR1 promotes the proliferative and migratory abilities of HNSCC cells, supporting its possible role as an oncogenic driver and therapeutic target.

### 3.15 Analysis of transcriptome sequencing following OLR1 gene knockdown

**Figure 15:**
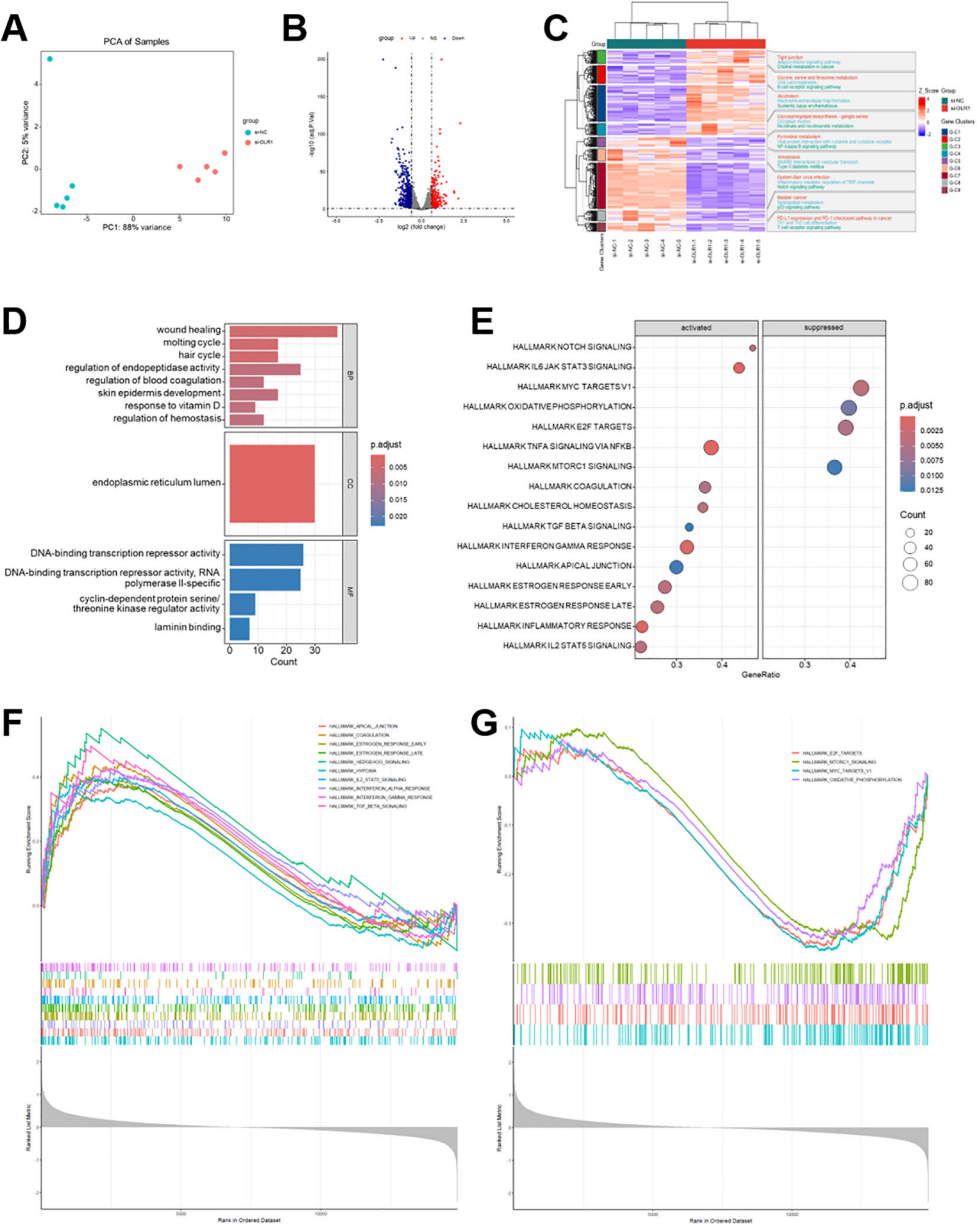
Transcriptomic sequencing analysis of OLR1 knockdown in HNSCC cells. **(A)** PCA illustrating distinct clustering between OLR1 knockdown (si-OLR1) and control (si-NC) groups. **(B)** The volcano plot displays DEGs with significantly upregulated (red) and downregulated (blue) genes. **(C)** Heatmap of hierarchical clustering of DEGs, indicating clear separation of expression patterns between groups. **(D)** Bar plots of GO enrichment analysis for BP, CC, and MF. **(E)** Dot plot of hallmark pathway enrichment analysis, with circle size representing gene count and color scale indicating adjusted *p*-value. **(F, G)** GSEA of hallmark gene sets, demonstrating significantly enriched pathways in activated **(F)** and suppressed **(G)** BP following OLR1 knockdown.

Transcriptome sequencing on SAS cells was conducted with OLR1 knockdown to investigate its impacts on downstream pathways. Principal component analysis (PCA) revealed a distinct separation between si-OLR1 and si-NC groups, highlighting significant transcriptomic alterations due to OLR1 knockdown (**Figure 15A**). A total of 298 genes were upregulated, while 470 genes were significantly downregulated, underscoring the broad effect of OLR1 on gene expression regulation (**Figure 15B**). Hierarchical clustering revealed marked downregulation of genes associated with PD-L1 expression and the PD-1 checkpoint pathway in cancer in the OLR1 knockdown group compared to the negative control group (**Figure 15C**). This cluster included pathways linked to Th1 and Th2 cell differentiation and T cell receptor signaling; all of them exhibited a similar suppression pattern following OLR1 silencing. The results of the GO analysis of DEGs are displayed in **Figure 15D**. GSEA revealed that OLR1 knockdown in SAS cells significantly activated hallmark pathways linked to tumor progression and immune regulation. **Figures 15E–G** illustrate that OLR1 silencing activates numerous anti-tumor immune pathways, such as interferon-γ response, IL6/JAK/STAT3 signaling, TNFα signaling via NF-κB, and inflammatory response. Simultaneously, characteristic pathways connected to tumor progression, such as MYC targets and mTORC1 signaling, were also significantly suppressed. These results, along with our earlier findings implicating OLR1 in promoting HNSCC growth and immune evasion, imply that OLR1 may contribute to tumor progression by maintaining oncogenic signaling and suppressing anti-tumor immunity.

## 4 Discussion

The heterogeneity of HNSCC tumors, characterized by differences in immune infiltration and genetic changes, complicates clinical management and emphasizes the urgent need for novel biomarkers and therapeutic targets to improve patient stratification and outcomes (27–29). This study investigated the prognostic importance and functional roles of Breg-related genes in HNSCC by using comprehensive bioinformatics analyses of TCGA-HNSCC and GEO datasets. A Breg-related gene signature was developed that effectively classified patients into different risk groups, revealing significant differences in outcomes, immune cell infiltration, response to immunotherapy, and sensitivity to anticancer agents. This study indicates that the signature of Breg-related genes is critical for predicting prognosis, impacting the tumor immune environment, and affecting drug response, thereby providing potential targets for personalized treatment strategies in HNSCC management.

To the best of our knowledge, this study establishes the first comprehensive prognostic signature specifically derived from Breg-related genes in HNSCC. While previous studies have developed immune-related prognostic models in HNSCC, such as those based on autophagy-related genes (30) or ADME-related genes (31), none have specifically delineated the impact of Breg-mediated immune suppression on patient outcomes in this context. Our 8-gene signature not only stratifies patients with robust accuracy across multiple cohorts but also reveals a distinct ‘immune-cold’ phenotype in high-risk patients, characterized by reduced immune infiltration and lower checkpoint expression. This finding provides a nuanced understanding of the HNSCC microenvironment that differs from general immune signatures, highlighting the specific role of Breg-associated pathways in driving immune evasion and poor prognosis in head and neck cancers.

The analyses in this study, which integrated Breg-related gene signatures with TMB and additional clinicopathological features, revealed that patients with high-risk Breg signatures frequently display unique mutational landscapes and that the combination of these molecular and clinicopathological parameters improves prognostic accuracy. The association between TMB and immunosuppressive cell populations such as Bregs remains unclear, despite prior research establishing that high TMB is associated with increased neoantigen load and, in certain situations, enhanced response to ICB (32–34). These results are consistent with recent studies in lung adenocarcinoma and bladder cancer, where signatures of Breg-related genes, when combined with TMB or other clinical variables in nomogram models, provide better prognostic discrimination than models that rely only on genetic or clinical criteria (11, 13). This integrative approach represents a methodological advancement over earlier HNSCC prognostic models that mostly relied on demographic, clinical, or epigenetic data without specifically considering Bregs (30).

WGCNA and GSEA analyses were conducted to investigate the mechanisms behind the prognostic difference between low- and high-risk patients, identifying a significant negative enrichment of immune-related pathways in high-risk individuals. Additional immune cell infiltration in response to Breg-derived risk stratification revealed a distinct pattern of immune cell paucity in patients with high-risk HNSCC. Decreased infiltration of activated B cells, Tregs, Th1/Th17 cells, and macrophages indicated a broad immune exclusion phenotype, which is consistent with the concept of “immune-cold” tumors described (11). According to research using an ADME-related gene signature, high-risk patients with HNSCC had markedly lower infiltration levels of immune cells, particularly B and T cells, which were associated with a poor prognosis and a reduced immunotherapy response (31). Additionally, other studies reinforce this finding, such as those in immune-related lncRNA classifications, where the C1 subtype (high-risk) was associated with a high proportion of inactive immune cells and immune cell deficiency (35). These results imply that the worse prognosis observed in patients with high-risk HNSCC may be attributed to immune suppression, emphasizing the possible interaction between Breg-related gene signature and TME.

The association between checkpoint molecule expression and risk groups in HNSCC was also examined, revealing that multiple checkpoint molecules, such as PD-1 and PD-L1, were downregulated in the high-risk group. The differences in immune cell infiltration and immune checkpoint expression among various risk groups led to the investigation of the potential of a signature for predicting immunotherapy responses. Recently, several studies have analyzed genomic data, immune microenvironment characteristics, radiological features, and biomarkers as potential predictors of response to ICB (36–40). This study examined three independent immunotherapy cohorts and calculated a risk score based on the signature for each patient. The results demonstrated that high-risk patients undergoing immunotherapy had significantly worse prognoses, indicating that the Breg-related signature is a reliable biomarker for predicting immunotherapy outcomes.

Furthermore, drug sensitivity analyses were performed across different risk groups of cancer cell lines. High-risk cells revealed markedly reduced IC_50_ values across various agents, with a strong negative correlation observed between the risk score and drug response. This correlation was particularly evident for Vincristine (microtubule inhibitor), Actinomycin D (anti-tumor antibiotic), Tamoxifen (selective estrogen receptor modulator), and Barasertib (Aurora B kinase inhibitor). These results underscore the possibility of risk score-based stratification to guide more precise and individualized therapeutic strategies for patients with HNSCC.

The focus was on additional experimental validation of OLR1, the most important gene identified by random forest analysis. The OLR1 gene, which codes for the oxidized low-density lipoprotein receptor 1, is of significant interest in tumor biology due to its role in lipid metabolism and inflammation (41–43). While OLR1 has been implicated in various malignancies (44, 45), its specific functions in HNSCC remain incompletely characterized.

Our pan-cancer and HNSCC-specific analyses confirmed that OLR1 is overexpressed in malignant tissues and predicts poor prognosis. Functional assays demonstrated that OLR1 silencing suppresses proliferation and migration of HNSCC cell lines. More importantly, transcriptomic profiling revealed that OLR1 knockdown markedly downregulates genes linked to PD-L1 expression, PD-1 checkpoint pathway, Th1/Th2 cell differentiation, and T cell receptor signaling, suggesting that OLR1 plays a crucial role in modulating immune checkpoint mechanisms and facilitating tumor immune evasion in HNSCC. These findings are consistent with previous studies showing that OLR1 expression in tumor-associated macrophages correlates with poor OS and immunosuppressive traits in HNSCC (46), and that OLR1 is associated with epithelial-mesenchymal transition and cuproptosis resistance (47). The therapeutic potential of targeting OLR1 is supported by drug sensitivity analyses identifying simvastatin and pazopanib as potential inhibitors (48), suggesting that pharmacological intervention against OLR1 may reverse immunosuppression and improve immunotherapy efficacy. Collectively, our results position OLR1 as a pivotal regulator integrating metabolic reprogramming, tumor progression, and immune checkpoint signaling in HNSCC, warranting further mechanistic investigation.

Beyond OLR1, the remaining seven genes in our signature also have documented roles in cancer progression and immune regulation. TGM2 (transglutaminase 2) is a critical regulator of the tumor microenvironment that promotes immune evasion and tumor progression across multiple cancer types (49, 50). It modulates adaptive immunity through NF-κB signaling and has been identified as a novel immune regulatory target (51, 52). TMC8, originally identified in HPV-related carcinogenesis (53), serves as a prognostic biomarker correlated with immune infiltration levels in various cancers including HNSCC (54, 55). CCL22, a macrophage-derived chemokine, plays a well-established immunosuppressive role by recruiting regulatory T cells to the tumor microenvironment through the CCL22/CCR4 axis (56, 57). This chemokine is secreted by both cancer cells and tumor-associated macrophages, contributing to immune tolerance (56, 58). GRIP2 has been identified as part of prognostic signatures in ovarian cancer and HNSCC, showing associations with immune infiltration and patient outcomes (59, 60). ZMAT1 functions as a tumor suppressor through the SIRT3/p53 signaling pathway, and its downregulation predicts poor prognosis in pancreatic ductal adenocarcinoma (61). FCRLA, an intracellular Fc receptor-like protein predominantly expressed in B cells, promotes malignant behavior and correlates with tumor immune infiltration in multiple cancers (62–64). SLC5A12, a sodium-coupled monocarboxylate transporter involved in lactate metabolism (65), serves as a prognostic marker in gastric cancer and is associated with tumor progression (66). Collectively, these genes represent diverse biological processes including immune modulation, metabolic reprogramming, and tumor progression, which together contribute to the robust prognostic performance of our Breg-related signature in HNSCC.

Although this study offers new insights, it still has limitations that should be acknowledged. The development and validation of the Breg-related gene signature were based solely on retrospective transcriptomic cohorts from the TCGA and GEO datasets, which may limit the applicability of these results to diverse real-world clinical environments with varied populations and treatment protocols. Second, functional experiments were performed exclusively in HNSCC cell lines. Although tumor-specific overexpression of OLR1 was consistently validated in tumor versus normal tissues across multiple public datasets, the absence of normal oral epithelial cell models limits the evaluation of cell-type–specific effects, which warrants further investigation in future studies. Additionally, while functional validation for OLR1 was conducted, the mechanistic roles of other signature genes remain inadequately investigated, limiting our understanding of the comprehensive biological functions and regulatory networks involving Bregs in HNSCC.

## 5 Conclusion

Overall, our results indicate that the Breg-related gene signature has significant prognostic value and associates with unique immune infiltration patterns and therapeutic responses in HNSCC. OLR1 identification as a contributor to tumor progression highlights the potential of targeting Breg-related pathways for translational applications. Future research should integrate prospective, multicenter clinical data and in-depth mechanistic studies of all signature genes to refine risk stratification models and improve the development of individualized immunotherapeutic strategies for patients with HNSCC.

## Data Availability

The datasets presented in this study can be found in online repositories. The names of the repository/repositories and accession number(s) can be found in the article/**Supplementary Material**.

## References

[1] Rahman S Kraljevic Pavelic S Markova-Car E . Circadian (de)regulation in head and neck squamous cell carcinoma. Int J Mol Sci. (2019) 20(11):2662. doi: 10.3390/ijms20112662. PMID: 31151182 PMC6600143

[2] Johnson DE Burtness B Leemans CR Lui VWY Bauman JE Grandis JR . Head and neck squamous cell carcinoma. Nat Rev Dis Primers. (2020) 6:92. doi: 10.1038/s41572-020-00224-3. PMID: 33243986 PMC7944998

[3] Gilardi M Saddawi-Konefka R Wu VH Lopez-Ramirez MA Wang Z Soto F . Microneedle-mediated intratumoral delivery of anti-Ctla-4 promotes Cdc1-dependent eradication of oral squamous cell carcinoma with limited Iraes. Mol Cancer Ther. (2022) 21:616–24. doi: 10.1158/1535-7163.MCT-21-0234. PMID: 35086958 PMC8983493

[4] Aboaid H Khalid T Hussain A Myat YM Nanda RK Srinivasmurthy R . Advances and challenges in immunotherapy in head and neck cancer. Front Immunol. (2025) 16:1596583. doi: 10.3389/fimmu.2025.1596583. PMID: 40547025 PMC12179090

[5] Hu C Liu M Li Y Zhao Y Sharma A Liu H . Recent advances and future perspectives of Car-T cell therapy in head and neck cancer. Front Immunol. (2023) 14:1213716. doi: 10.3389/fimmu.2023.1213716. PMID: 37457699 PMC10346844

[6] Sangal NR Tapescu I Kaki P Brody RM Carey RM . Immunotherapy utilization outcomes in distantly metastatic head and neck squamous cell carcinoma. JAMA Otolaryngol Head Neck Surg. (2025) 151:699–709. doi: 10.1001/jamaoto.2025.1351. PMID: 40471563 PMC12142472

[7] Jubran MR Vilenski D Flashner-Abramson E Shnaider E Vasudevan S Rubinstein AM . Overcoming resistance to Egfr monotherapy in Hnscc by identification and inhibition of individualized cancer processes. Theranostics. (2022) 12:1204–19. doi: 10.7150/thno.64347. PMID: 35154483 PMC8771558

[8] Catalan D Mansilla MA Ferrier A Soto L Oleinika K Aguillon JC . Immunosuppressive mechanisms of regulatory B cells. Front Immunol. (2021) 12:611795. doi: 10.3389/fimmu.2021.611795. PMID: 33995344 PMC8118522

[9] Rosser EC Mauri C . Regulatory B cells: origin, phenotype, and function. Immunity. (2015) 42:607–12. doi: 10.1016/j.immuni.2015.04.005. PMID: 25902480

[10] Choi JK Egwuagu CE . Interleukin 35 regulatory B cells. J Mol Biol. (2021) 433:166607. doi: 10.1016/j.jmb.2020.07.019. PMID: 32755620 PMC7779660

[11] Zhang L Zeng J Zhang X Zhang M Lin Y Lai F . Comprehensive analysis of regulatory B cell related genes in prognosis and therapeutic response in lung adenocarcinoma. Front Immunol. (2025) 16:1595408. doi: 10.3389/fimmu.2025.1595408. PMID: 40808964 PMC12343544

[12] Moreira H Dobosz A Cwynar-Zajac L Nowak P Czyzewski M Barg M . Unraveling the role of Breg cells in digestive tract cancer and infectious immunity. Front Immunol. (2022) 13:981847. doi: 10.3389/fimmu.2022.981847. PMID: 36618354 PMC9816437

[13] Zhou J Zhou R Zhu Y Deng S Muhuitijiang B Li C . Investigating the impact of regulatory B cells and regulatory B cell-related genes on bladder cancer progression and immunotherapeutic sensitivity. J Exp Clin Cancer Res. (2024) 43:101. doi: 10.1186/s13046-024-03017-8. PMID: 38566204 PMC10985985

[14] Jing Y Xu F Liang W Liu J Zhang L . Role of regulatory B cells in gastric cancer: latest evidence and therapeutics strategies. Int Immunopharmacol. (2021) 96:107581. doi: 10.1016/j.intimp.2021.107581. PMID: 33812259

[15] Chi H Zhao S Yang J Gao X Peng G Zhang J . T-cell exhaustion signatures characterize the immune landscape and predict Hcc prognosis via integrating single-cell Rna-seq and bulk Rna-sequencing. Front Immunol. (2023) 14:1137025. doi: 10.3389/fimmu.2023.1137025. PMID: 37006257 PMC10050519

[16] Wilkerson MD Hayes DN . Consensusclusterplus: a class discovery tool with confidence assessments and item tracking. Bioinformatics. (2010) 26:1572–3. doi: 10.1093/bioinformatics/btq170. PMID: 20427518 PMC2881355

[17] Yoshihara K Shahmoradgoli M Martinez E Vegesna R Kim H Torres-Garcia W . Inferring tumour purity and stromal and immune cell admixture from expression data. Nat Commun. (2013) 4:2612. doi: 10.1038/ncomms3612. PMID: 24113773 PMC3826632

[18] Hanzelmann S Castelo R Guinney J . Gsva: gene set variation analysis for microarray and Rna-seq data. BMC Bioinf. (2013) 14:7. doi: 10.1186/1471-2105-14-7. PMID: 23323831 PMC3618321

[19] Sturm G Finotello F List M . Immunedeconv: an R package for unified access to computational methods for estimating immune cell fractions from bulk Rna-sequencing data. Methods Mol Biol. (2020) 2120:223–32. doi: 10.1007/978-1-0716-0327-7_16. PMID: 32124323

[20] Li Y Ge X Peng F Li W Li JJ . Exaggerated false positives by popular differential expression methods when analyzing human population samples. Genome Biol. (2022) 23:79. doi: 10.1186/s13059-022-02648-4. PMID: 35292087 PMC8922736

[21] Xu S Hu E Cai Y Xie Z Luo X Zhan L . Using Clusterprofiler to characterize multiomics data. Nat Protoc. (2024) 19:3292–320. doi: 10.1038/s41596-024-01020-z. PMID: 39019974

[22] Friedman J Hastie T Tibshirani R . Regularization paths for generalized linear models via coordinate descent. J Stat Softw. (2010) 33:1–22. doi: 10.18637/jss.v033.i01 20808728 PMC2929880

[23] Mayakonda A Lin DC Assenov Y Plass C Koeffler HP . Maftools: efficient and comprehensive analysis of somatic variants in cancer. Genome Res. (2018) 28:1747–56. doi: 10.1101/gr.239244.118. PMID: 30341162 PMC6211645

[24] Langfelder P Horvath S . Wgcna: an R package for weighted correlation network analysis. BMC Bioinf. (2008) 9:559. doi: 10.1186/1471-2105-9-559. PMID: 19114008 PMC2631488

[25] Ren X Yao XR Chen K Xiao WT He JY . Crispr-Cas9 screening identifies a gene signature predictive of prognosis in glioblastoma. Sci Rep. (2025) 15:21077. doi: 10.1038/s41598-025-07815-8. PMID: 40594722 PMC12216265

[26] Pang KL Li P Yao XR Xiao WT Ren X He JY . Deciphering a proliferation-essential gene signature based on Crispr-Cas9 screening to predict prognosis and characterize the immune microenvironment in Hnscc. BMC Cancer. (2025) 25:756. doi: 10.1186/s12885-025-14181-1. PMID: 40264050 PMC12016166

[27] Huang X Duijf PHG Sriram S Perera G Vasani S Kenny L . Circulating tumour DNA alterations: emerging biomarker in head and neck squamous cell carcinoma. J BioMed Sci. (2023) 30:65. doi: 10.1186/s12929-023-00953-z. PMID: 37559138 PMC10413618

[28] Li Y Lu C . Targeting epigenetic dysregulations in head and neck squamous cell carcinoma. J Dent Res. (2025) 104:225–34. doi: 10.1177/00220345241297122. PMID: 39698794 PMC12764359

[29] Jiang B Elkashif A Coulter JA Dunne NJ McCarthy HO . Immunotherapy for Hpv negative head and neck squamous cell carcinoma. Biochim Biophys Acta Rev Cancer. (2024) 1879:189138. doi: 10.1016/j.bbcan.2024.189138. PMID: 38889878

[30] Xu Z Chen X Song X Kong X Chen J Song Y . Athena: an independently validated autophagy-related epigenetic prognostic prediction model of head and neck squamous cell carcinoma. Clin Epigenet. (2023) 15:97. doi: 10.1186/s13148-023-01501-0. PMID: 37296474 PMC10257287

[31] Tang X Li R Wu D Wang Y Zhao F Lv R . Development and validation of an Adme-related gene signature for survival, treatment outcome and immune cell infiltration in head and neck squamous cell carcinoma. Front Immunol. (2022) 13:905635. doi: 10.3389/fimmu.2022.905635. PMID: 35874705 PMC9304892

[32] Hanrahan GB Giobbie-Hurder A Allais B Vogelzang J Fay C Tsibris HC . Melanoma tumor mutational burden and indoor tanning exposure. JAMA Dermatol. (2025) 161:198–202. doi: 10.1001/jamadermatol.2024.4819. PMID: 39661348 PMC11840639

[33] Merchant M Ranjan A Pang Y Yu G Kim O Khan J . Tumor mutational burden and immunotherapy in gliomas. Trends Cancer. (2021) 7:1054–8. doi: 10.1016/j.trecan.2021.08.005. PMID: 34580037 PMC10423405

[34] Zheng M . Tumor mutation burden for predicting immune checkpoint blockade response: the more, the better. J Immunother Cancer. (2022) 10(1):e003087. doi: 10.1136/jitc-2021-003087. PMID: 35101940 PMC8804687

[35] Cao R Cui L Zhang J Ren X Cheng B Xia J . Immune-related Lncrna classification of head and neck squamous cell carcinoma. Cancer Cell Int. (2022) 22:25. doi: 10.1186/s12935-022-02450-z. PMID: 35033066 PMC8760760

[36] Valero C Golkaram M Vos JL Xu B Fitzgerald C Lee M . Clinical-genomic determinants of immune checkpoint blockade response in head and neck squamous cell carcinoma. J Clin Invest. (2023) 133(19):e169823. doi: 10.1172/JCI169823. PMID: 37561583 PMC10541199

[37] Sun J Fang G Zuo Z Yu X Xue L Li C . Identification of immune subtypes for predicting the prognosis of patients in head and neck squamous cell carcinoma. Technol Cancer Res Treat. (2021) 20:15330338211045823. doi: 10.1177/15330338211045823. PMID: 34657509 PMC8521413

[38] Ruiz-Torres DA Bryan ME Hirayama S Merkin RD Luciani E Roberts TJ . Spatial characterization of tertiary lymphoid structures as predictive biomarkers for immune checkpoint blockade in head and neck squamous cell carcinoma. Oncoimmunology. (2025) 14:2466308. doi: 10.1080/2162402X.2025.2466308. PMID: 39963988 PMC11845054

[39] Chang TG Spathis A Schaffer AA Gavrielatou N Kuo F Jia D . Tumor and blood B-cell abundance outperforms established immune checkpoint blockade response prediction signatures in head and neck cancer. Ann Oncol. (2025) 36:309–20. doi: 10.1016/j.annonc.2024.11.008. PMID: 39551185 PMC11845298

[40] Sridharan V Rahman RM Huang RY Chau NG Lorch JH Uppaluri R . Radiologic predictors of immune checkpoint inhibitor response in advanced head and neck squamous cell carcinoma. Oral Oncol. (2018) 85:29–34. doi: 10.1016/j.oraloncology.2018.08.005. PMID: 30220316

[41] Mu J Li J Chen Z Chen Y Lin Q Zhang L . Rice bran peptides target lectin-like oxidized low-density lipoprotein receptor-1 to ameliorate atherosclerosis. Food Funct. (2025) 16:867–84. doi: 10.1039/d4fo04514a. PMID: 39636043

[42] Munno M Mallia A Greco A Modafferi G Banfi C Eligini S . Radical oxygen species, oxidized low-density lipoproteins, and lectin-like oxidized low-density lipoprotein receptor 1: a vicious circle in atherosclerotic process. Antioxidants (Basel). (2024) 13(5):583. doi: 10.3390/antiox13050583. PMID: 38790688 PMC11118168

[43] Bing T Shanlin X Jisheng W Jie H Ruichao C Zhiwei Z . Dysregulated lipid metabolism and intervertebral disc degeneration: the important role of Ox-Ldl/Lox-1 in endplate chondrocyte senescence and calcification. Mol Med. (2024) 30:117. doi: 10.1186/s10020-024-00887-8. PMID: 39123116 PMC11311918

[44] Sun X Fu X Xu S Qiu P Lv Z Cui M . Olr1 is a prognostic factor and correlated with immune infiltration in breast cancer. Int Immunopharmacol. (2021) 101:108275. doi: 10.1016/j.intimp.2021.108275. PMID: 34688153

[45] Yang G Xiong G Feng M Zhao F Qiu J Liu Y . Olr1 promotes pancreatic cancer metastasis via increased C-Myc expression and transcription of Hmga2. Mol Cancer Res. (2020) 18:685–97. doi: 10.1158/1541-7786.MCR-19-0718. PMID: 32019809

[46] Zhang P Zhao Y Xia X Mei S Huang Y Zhu Y . Expression of Olr1 gene on tumor-associated macrophages of head and neck squamous cell carcinoma, and its correlation with clinical outcome. Oncoimmunology. (2023) 12:2203073. doi: 10.1080/2162402X.2023.2203073. PMID: 37089448 PMC10120517

[47] Wu L Liu Y Deng W Wu T Bu L Chen L . Olr1 is a pan-cancer prognostic and immunotherapeutic predictor associated with Emt and cuproptosis in Hnscc. Int J Mol Sci. (2023) 24(16):12904. doi: 10.3390/ijms241612904. PMID: 37629087 PMC10454104

[48] He D Yang Z Zhang T Luo Y Peng L Yan J . Multi-omics and machine learning-driven Cd8(+) T cell heterogeneity score for head and neck squamous cell carcinoma. Mol Ther Nucleic Acids. (2025) 36:102413. doi: 10.1016/j.omtn.2024.102413. PMID: 40027882 PMC11869859

[49] Waller EK Chaudagar K . Pancreatic cancer cell-intrinsic transglutaminase-2 promotes T cell suppression through microtubule-dependent secretion of immunosuppressive cytokines. J Immunother Cancer. (2025) 13(5):e012188. doi: 10.1136/jitc-2025-012188. PMID: 40447317 PMC12128397

[50] Caksa S Purwin TJ Erkes DA DeRosa KM Kitterman E Barnada SM . Elevated transglutaminase-2 in Sox10-deficient melanoma promotes tumor onset and decreases intratumoral Cd4+ T cells. Cancer Res. (2025) 85:3614–32. doi: 10.1158/0008-5472.CAN-24-3267. PMID: 40742313 PMC12398951

[51] Marquardt V Theruvath J Pauck D Picard D Qin N Blumel L . Tacedinaline (Ci-994), a class I Hdac inhibitor, targets intrinsic tumor growth and leptomeningeal dissemination in Myc-driven medulloblastoma while making them susceptible to anti-Cd47-induced macrophage phagocytosis via Nf-Kb-Tgm2 driven tumor inflammation. J Immunother Cancer. (2023) 11(1):e005871. doi: 10.1136/jitc-2022-005871. PMID: 36639156 PMC9843227

[52] Lan K April V Jamali F Sabri S Abdulkarim B . Current insights on transglutaminase 2: Exploring its functions, mechanisms, and therapeutic potential in glioblastoma. Crit Rev Oncol Hematol. (2025) 214:104894. doi: 10.1016/j.critrevonc.2025.104894. PMID: 40818831

[53] Rehm TM Parpoulas C Straub E Iftner T Stubenrauch F . No evidence for restriction of beta-Hpv8 gene expression by epidermodysplasia verruciformis susceptibility genes Cib1, Tmc6, or Tmc8 in keratinocytes. Tumour Virus Res. (2025) 20:200328. doi: 10.1016/j.tvr.2025.200328. PMID: 41135646 PMC12617823

[54] Song J Tang Y Luo X Shi X Song F Ran L . Pan-cancer analysis reveals the signature of Tmc family of genes as a promising biomarker for prognosis and immunotherapeutic response. Front Immunol. (2021) 12:715508. doi: 10.3389/fimmu.2021.715508. PMID: 34899684 PMC8660091

[55] Liu F Qin Y Luo W Ruan X Lu L Feng B . Construction of a risk model associated with tryptophan metabolism and identification of related molecular subtypes in laryngeal squamous cell carcinoma. Front Genet. (2025) 16:1530334. doi: 10.3389/fgene.2025.1530334. PMID: 40196225 PMC11973366

[56] Song NY Li X Ma B Willette-Brown J Zhu F Jiang C . Ikkalpha-deficient lung adenocarcinomas generate an immunosuppressive microenvironment by overproducing Treg-inducing cytokines. Proc Natl Acad Sci USA. (2022) 119:1530334. doi: 10.1073/pnas.2120956119. PMID: 35121655 PMC8833198

[57] Hao X Liu L Li S Wang Q Xu Z Liu X . The regulatory role and mechanism of Tgfbi on Tregs in the immune microenvironment of clear cell renal cell carcinoma. Int J Surg. (2025) 111:9134–46. doi: 10.1097/JS9.0000000000003225. PMID: 40865943 PMC12695357

[58] Zhang K Liu J Liu Q Zhu N Ye B . Macrophage-derived chemokines in T cell regulation: Implications for cancer immunotherapy. Front Immunol. (2025) 16:1739154. doi: 10.3389/fimmu.2025.1739154. PMID: 41635851 PMC12861892

[59] Lei H Guo K Shu G Wang M Li Y Tan Z . Gjb2 as a novel prognostic biomarker associated with immune infiltration and cuproptosis in ovarian cancer. Apoptosis. (2025) 30:1589–613. doi: 10.1007/s10495-025-02119-8. PMID: 40375037 PMC12167356

[60] Zhou S Jiang Y Xiong P Li Z Jia L Yuan W . Identification and prognostic analysis of propionate metabolism-related genes in head and neck squamous cell carcinoma. Front Oncol. (2025) 15:1518587. doi: 10.3389/fonc.2025.1518587. PMID: 40575157 PMC12198198

[61] Ma Z Li Z Wang S Zhou Z Liu C Zhuang H . Zmat1 acts as a tumor suppressor in pancreatic ductal adenocarcinoma by inducing Sirt3/P53 signaling pathway. J Exp Clin Cancer Res. (2022) 41:130. doi: 10.1186/s13046-022-02310-8. PMID: 35392973 PMC8988381

[62] Liu JP Jiang YF Liu JW Tian CJ Lin YZ Yang YZ . Fc receptor-like a promotes Malignant behavior in renal cell carcinoma and correlates with tumor immune infiltration. Cancer Med. (2024) 13:e70072. doi: 10.1002/cam4.70072. PMID: 39108036 PMC11303447

[63] Liu Y Chen Y Hu X Meng J Li X . Development and validation of the B cell-associated Fc receptor-like molecule-based prognostic signature in skin cutaneous melanoma. BioMed Res Int. (2020) 2020:8509805. doi: 10.1155/2020/8509805. PMID: 32908921 PMC7463385

[64] Liu X Shang X Li J Zhang S . The prognosis and immune checkpoint blockade efficacy prediction of tumor-infiltrating immune cells in lung cancer. Front Cell Dev Biol. (2021) 9:707143. doi: 10.3389/fcell.2021.707143. PMID: 34422829 PMC8370893

[65] Certo M Pontarini E Gilbert SG Schmidt R Turner JD Lucchesi D . Lactate signalling leads to aggregation of immune-inflammatory hotspots and Slc5a12 blockade promotes their resolution. Nat Metab. (2025) 7:1663–80. doi: 10.1038/s42255-025-01331-9. PMID: 40759752 PMC12373510

[66] Lin C Ye J Xu C Zheng Y Xu Y Chen Y . Evaluating lactate metabolism for prognostic assessment and therapy response prediction in gastric cancer with emphasis on the oncogenic role of Slc5a12. Biochim Biophys Acta Gen Subj. (2025) 1869:130739. doi: 10.1016/j.bbagen.2024.130739. PMID: 39672477

