# Supplementary material for: Regulatory B cell-related gene signature predicts prognosis and immune landscape in head and neck squamous cell carcinoma
Source: Front Immunol. 2026 Apr 10;17:1739076. doi: 10.3389/fimmu.2026.1739076 (PMC13106120; doi:10.3389/fimmu.2026.1739076)
Supplement: Supplementary file 1 [file Supplementaryfile1.docx]

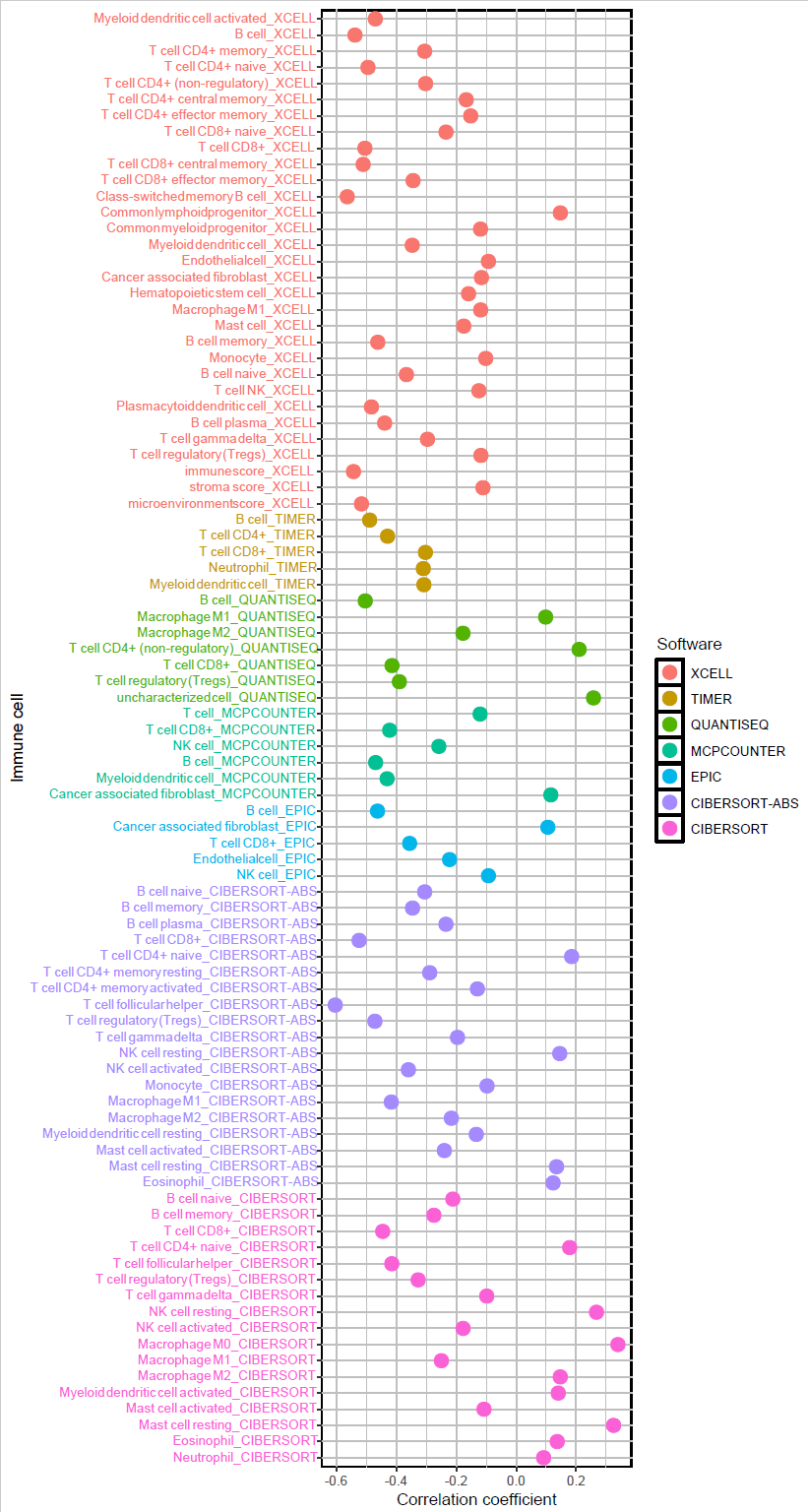

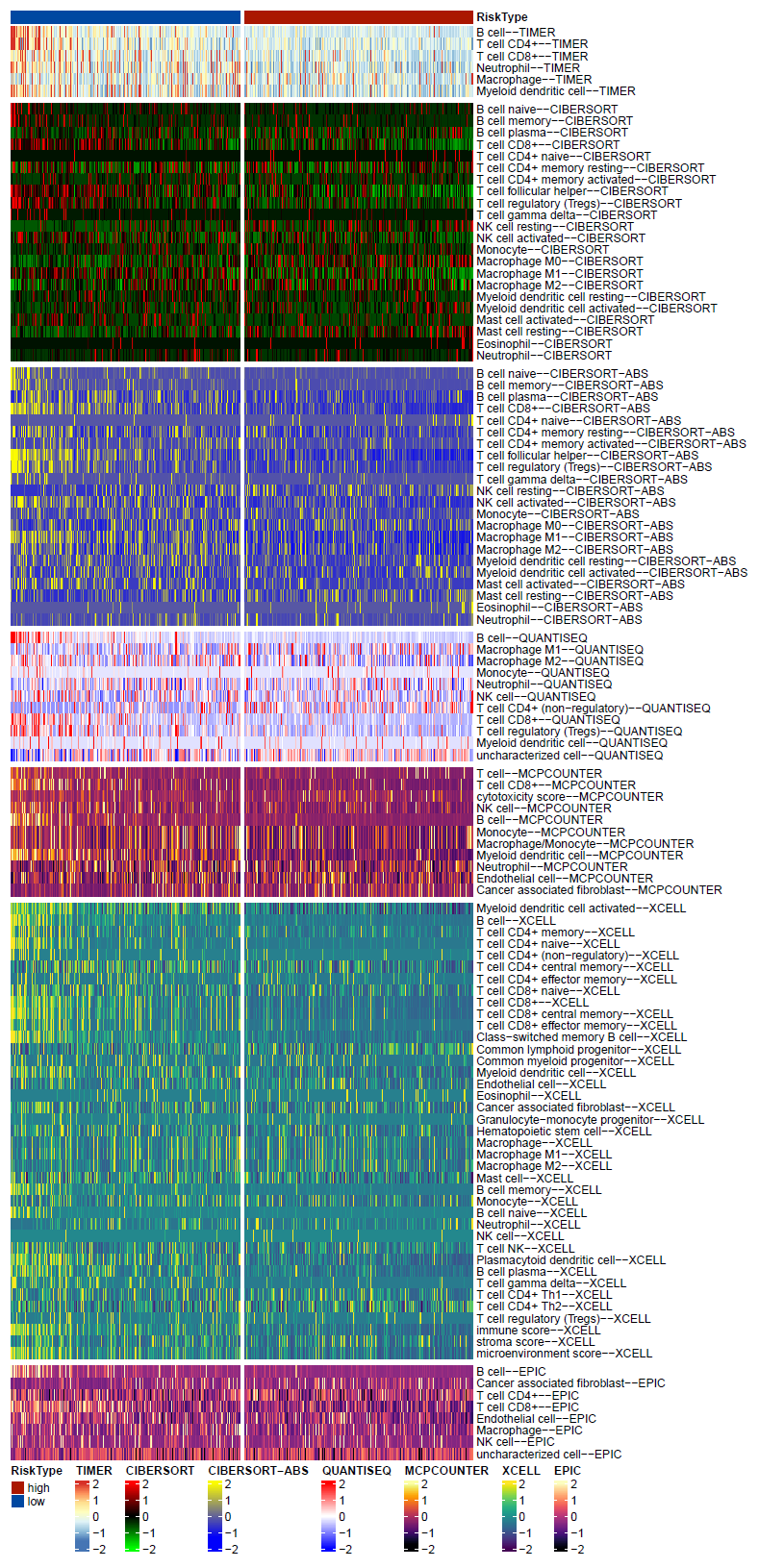


**A**

**B**

**Figure S1. Validation of immune infiltration patterns using multiple deconvolution algorithms.**

(A) Correlation analysis between risk score and the abundance of immune cell subtypes across six different deconvolution algorithms (XCell, TIMER, QUANTISEQ, MCPCOUNTER, CIBERSORT-ABS, CIBERSORT). (B) Heatmaps illustrating the relative abundance of immune cell subsets in low-risk (blue) versus high-risk (red) HNSCC patients, as estimated by each of the six deconvolution algorithms.
