# Supplementary material for: Regulatory B cell-related gene signature predicts prognosis and immune landscape in head and neck squamous cell carcinoma
Source: Front Immunol. 2026 Apr 10;17:1739076. doi: 10.3389/fimmu.2026.1739076 (PMC13106120; doi:10.3389/fimmu.2026.1739076)
Supplement: Supplementary file 2 [file Table1.docx]

| Variable | Overall (n = 493) |
| --- | --- |
| Age, median (range) | 61 (19-88) |
| Gender, n (%) | Male: 363 (73.6%)  Female: 130 (26.4%) |
| Follow-up time, median (days) | 643 (30-6417) |
| Clinical T stage, n (%) | T1-2: 175 (35.5%)  T3-4: 304 (61.7%)  Unknown: 14 (2.8%) |
| Clinical N stage, n (%) | N0: 236 (47.9%)  N1-3: 236 (47.9%)  Unknown: 21 (4.2%) |
| Clinical M stage, n (%) | M0: 464 (94.1%)  M1: 5 (1.0%)  Unknown: 24 (4.9%) |
| Clinical stage, n (%) | I-II: 113 (22.9%)  III-IV: 367 (74.4%)  Unknown: 13 (2.7%) |
| Pathologic T stage, n (%) | T1-2: 176 (35.7%)  T3-4: 262 (53.1%)  Unknown: 55 (11.2%) |
| Pathologic N stage, n (%) | N0: 167 (33.9%)  N1-3: 233 (47.3%)  Unknown: 93 (18.8%) |
| Pathologic M stage, n (%) | M0: 181 (36.7%)  M1: 1 (0.2%)  Unknown: 311 (63.1%) |
| Pathologic stage, n (%) | I-II: 94 (19.1%)  III-IV: 331 (67.1%)  Unknown: 68 (13.8%) |
| Lymphovascular invasion, n (%) | Negative: 216 (43.8%)  Positive: 117 (23.7%)  Unknown: 160 (32.5%) |
| Perineural invasion, n (%) | Negative: 183 (37.1%)  Positive: 163 (33.1%)  Unknown: 147 (29.8%) |
| Tumor status, n (%) | Tumor free: 312 (63.3%)  With tumor: 137 (27.8%)  Unknown: 44 (8.9%) |
| Vital status, n (%) | Alive: 333 (67.5%)  Dead: 160 (32.5%) |

**Table S1. Baseline characteristics of the TCGA-HNSCC cohort.**
