# Supplementary material for: Regulatory B cell-related gene signature predicts prognosis and immune landscape in head and neck squamous cell carcinoma
Source: Front Immunol. 2026 Apr 10;17:1739076. doi: 10.3389/fimmu.2026.1739076 (PMC13106120; doi:10.3389/fimmu.2026.1739076)
Supplement: Supplementary file 5 [file Table4.docx]

| **Gene** | **TCGA-train**  **(Foldchange and p value)** | **TCGA-test**  **(Foldchange and p value)** | **TCGA-entire**  **(Foldchange and p value)** | **GSE41613**  **(Foldchange and p value)** | **GSE65858**  **(Foldchange and p value)** | **Trend**  **(high- vs. low-risk groups)** |
| --- | --- | --- | --- | --- | --- | --- |
| OLR1 | 1.6615095  (7.942155e-04) | 1.6531121  (3.360671e-04) | 1.6510564  (1.690178e-06) | 3.4391388  (7.566747e-09) | 1.5651490  (2.963197e-20) | Risk |
| SLC5A12 | 2.9847821  (3.050129e-12) | 2.5518115  (6.600973e-10) | 2.7622527  (1.296445e-20) | 1.0839080  (7.679003e-02) | 1.0459399  (1.823252e-04) | Risk |
| TGM2 | 1.3103710  (1.939949e-02) | 1.5022803  (5.881821e-03) | 1.3813407  (3.734484e-04) | 2.2588595  (2.989764e-04) | 1.3273511  (3.098426e-07) | Risk |
| CCL22 | 0.4823631  (1.847154e-11) | 0.5085474  (1.732287e-09) | 0.4948526  (4.972287e-20) | 0.8170551  (1.416916e-03) | 0.8554171  (8.573565e-05) | Protective |
| TMC8 | 0.5110817  (8.993180e-13) | 0.6134620  (5.897799e-10) | 0.5602399  (1.018822e-20) | 0.6531676  (2.824997e-04) | 0.9836384  (1.392383e-02) | Protective |
| ZMAT1 | 0.5898327  (1.597806e-14) | 0.5424886  (3.716209e-12) | 0.5776369  (5.659828e-26) | 0.9219192  (7.398391e-04) | 0.9897939  (8.192803e-02) | Protective |
| FCRLA | 0.6203497  (1.091980e-12) | 0.5300419  (1.122976e-10) | 0.5934953  (6.275317e-22) | 0.8224884  (1.288290e-04) | 0.7689171  (4.811268e-08) | Protective |
| GRIP2 | 0.5998129  (8.089229e-12) | 0.6544211  (5.615607e-05) | 0.6057934  (5.318845e-14) | 1.0029839  (6.208719e-01) | 0.9119648  (2.204000e-09) | Protective |

**Table S4.**

**Expression differences of the eight Breg-related signature genes between high- and low-risk groups across cohorts (Wilcoxon rank-sum test).**
